# New pan-ALK inhibitor-resistant EML4::ALK mutations detected by liquid biopsy in lung cancer patients

**DOI:** 10.1038/s41698-024-00498-w

**Published:** 2024-03-06

**Authors:** Matteo Villa, Federica Malighetti, Elisa Sala, Geeta G. Sharma, Giulia Arosio, Maria Gemelli, Chiara Manfroni, Diletta Fontana, Nicoletta Cordani, Raffaella Meneveri, Alfonso Zambon, Rocco Piazza, Fabio Pagni, Diego Cortinovis, Luca Mologni

**Affiliations:** 1 Department of Medicine and Surgery, University of Milano-Bicocca, Monza, Italy; 2 SC Medical Oncology, Fondazione IRCCS San Gerardo dei Tintori, Monza, Italy; 3 Medical Oncology Unit, Istituto di Ricovero e Cura a Carattere Scientifico (IRCCS) MultiMedica, Milan, Italy; 4 Department of Chemistry and Geological Sciences, University of Modena and Reggio Emilia, Modena, Italy; 5 Department of Pathology, Fondazione IRCCS San Gerardo dei Tintori, Monza, Italy

**Keywords:** Cancer, Cancer genetics

## Abstract

ALK and ROS1 fusions are effectively targeted by tyrosine kinase inhibitors (TKIs), however patients inevitably relapse after an initial response, often due to kinase domain mutations. We investigated circulating DNA from TKI-relapsed NSCLC patients by deep-sequencing. New EML4::ALK substitutions, L1198R, C1237Y and L1196P, were identified in the plasma of NSCLC ALK patients and characterized in a Ba/F3 cell model. Variants C1237Y and L1196P demonstrated pan-inhibitor resistance across 5 clinical and 2 investigational TKIs.

Translocations of the Anaplastic Lymphoma Kinase (*ALK*) gene represent an actionable genomic alteration in 5–8% of Non-Small Cell Lung Cancer (NSCLC) patients^1^. Several tyrosine kinase inhibitors (TKIs) are currently in use for advanced ALK + NSCLC^2^. Despite great efficacy, resistance remains an issue, as virtually all tumors eventually relapse leading to patients death^3,4^. A smaller fraction (1–3%) of NSCLC patients carry ROS1 fusions, that can be treated with some ALK TKIs^5^.

Analysis of circulating tumor DNA (ctDNA) can improve prognostic and predictive capabilities, by allowing early detection of relapse and drug-resistant mutations^6,7^, and by collecting information from all metastatic sites, yielding a more comprehensive picture of the mutational landscape of the heterogeneous tumor cell population, compared to tissue biopsies. We previously described a brigatinib-resistant lung cancer patient carrying a compound L1196M/G1202R ALK mutation^8^. Here, we report an extended series of 15 consecutive relapsed NSCLC patients (12 ALK+, 3 ROS1+) treated at our center that were investigated by liquid biopsy at TKI failure, through amplicon deep sequencing of the *ALK/ROS1* kinase domains. Novel *ALK* mutations, including a pan-drug resistant mutant located at the αE-helix (C1237Y), a novel L1198R substitution and an unusual gatekeeper variant (L1196P), were identified and studied in the Ba/F3 cell system to confirm their functional role in clinical relapse. No mutation was called in healthy samples using the parameters set for the analysis.

The study enrolled 15 consecutive patients affected by ALK+ or ROS1 + NSCLC, relapsing on TKI monotherapy at any line of treatment (Table 1 and Fig. 1a). Five patients received an ALK inhibitor as first-line therapy, the remaining 10 patients had relapsed on chemotherapy. Patients’ characteristics are reported in Table 1. Plasma was collected at first relapse after enrolment and, only in 3 cases (patients n. 3, 13 and 14), a second plasma sample was obtained at the time of next-line TKI failure. For one patient (n. 4), a second blood draw was collected during treatment beyond progression (TBP). Plasma ctDNA analysis detected an ALK mutation in 7/18 [39%] relapse samples from 7/15 (47%) patients (Fig. 1b), in line with published data^7^. Mutations were more frequent after progression on second-generation compounds (50% mutated samples) than after crizotinib (17%), as reported^9^, and mutation frequency increased with increasing lines of previous TKI therapy (22% *vs* 56% mutated samples after progression on first TKI and after >1 TKI, respectively; Fig. 1c and Supplementary Table 1). Progression-free survival tended to be shorter when TKI treatment selected an ALK mutation (Fig. 1d, e), however this may be biased by the higher frequency of mutants in advanced lines of therapy. Overall survival was not different between mutated and non-mutated patients. Among 12 patients carrying an EML4::ALK fusion, 10 variants potentially driving resistance were found (Fig. 2a). Mutations were introduced in Ba/F3 cells to validate their effects on drug sensitivity. No ROS1 mutations were found in 3 CD74::ROS1 fusion-positive patients.

**Table 1:** Patients’ characteristics, treatments and mutations.

Plasma was obtained at first relapse after enrolment and, in 3 cases (patients n. 3, 13 and 14), a second plasma sample was collected at the time of next-line TKI failure. For one patient (n. 4), a second blood draw was obtained during treatment beyond progression (TBP). In total, 19 plasma samples from 18 relapses (9 at first TKI and 9 with previous exposure to one or more TKIs) and 1 TBP, were analysed. Two time points of plasma collection from the same patient are separated by a dotted line.

*CDDP* Cisplatin, *PEM* Pemetrexed, *GEM* Gemcitabine, *CARB* Carboplatin, *PMB* Pembrolizumab, *VAF* variant allele frequency.

^1^Treatment beyond progression.

^2^A different variant at the same position is reported.

**Fig. 1:**
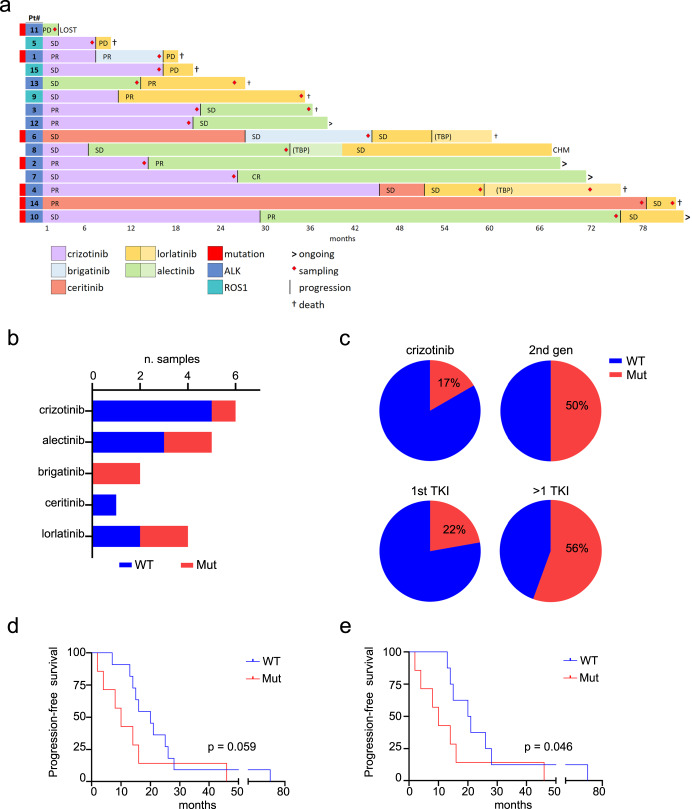
Patients’ clinical data. **a** Swimmer plot showing patients’ treatment and response, sorted by time on treatment. Pre-TKI therapy is not shown. A red diamond indicates the time of sampling. Bar colors represent TKIs, as indicated in the legend. TBP treatment beyond progression, PR partial response, SD stable disease, PD progressive disease, CHM chemotherapy, † death. **b** Number of wild-type (WT) and mutated (Mut) samples from subjects progressing on each inhibitor, excluding the one TBP sample. **c** Pie charts indicate the frequency of WT and Mut samples after crizotinib *vs* second-generation drugs (*top*) and after 1 *vs* > 1 TKI (*bottom*). Progression-free survival of WT *vs* Mut cases, considering the whole cohort (**d**) or ALK+ patients only (**e**).

**Fig. 2:**
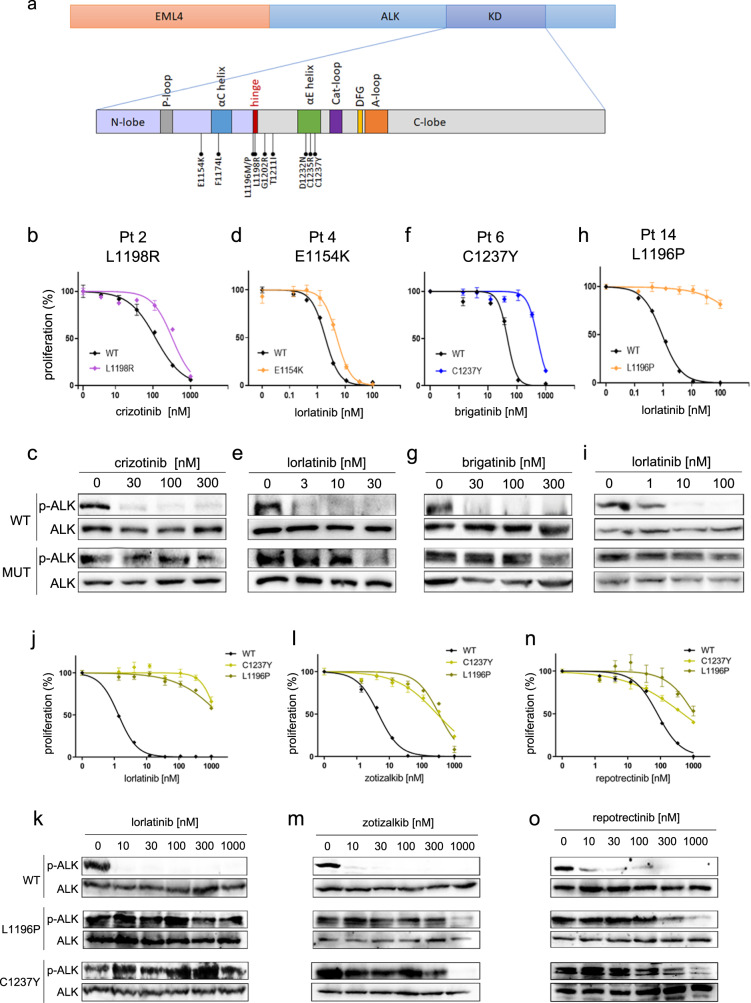
Effect of mutations on drug sensitivity. **a** Diagram representing the EML4::ALK fusion, with a zoom on the kinase domain (KD), in which structural motifs are indicated. Mutations identified in this study are shown. **b–i** Cell viability and EML4::ALK phosphorylation of Ba/F3 cells expressing wild-type (WT, black lines) or mutant (colored lines) EML4::ALK, treated with the indicated TKIs. **b**, **c** L1198R from patient 2; **d**, **e** E1154K from patient 4; **f**, **g** C1237Y from patient 6; **h**, **i** L1196P from patient 14. (**j–o**) L1196P and C1237Y variants were tested with lorlatinib (**j**, **k**), zotizalkib (**l**, **m**) and repotrectinib (**n**, **o**). All data points represent mean ± s.e.m. Phospho-ALK and total ALK blots were run on the same membrane, after stripping.

Patient 1, carrying a compound L1196M/G1202R mutation conferring high resistance to all drugs, was reported previously^8^. Patient 2, relapsed on crizotinib, carried a novel L1198R variant; when expressed in Ba/F3 cells, EML4::ALK^L1198R^ was resistant to crizotinib in cell growth assays (Fig. 2b) and by ALK phosphorylation (Fig. 2c), possibly explaining the relapse. The mutation provided moderate resistance also to other TKIs (Table 2, Supplementary Fig. 1 and Supplementary Fig. 2).

**Table 2:** Activity of ALK inhibitors (IC_50_, nM) on EML4::ALK-transfected Ba/F3 cells, wild-type (WT) or mutant.

IC_50_ [nM]	Crizotinib	Brigatinib	Ceritinib	Alectinib	Lorlatinib	Zotizalkib	Repotrectinib
WT	90	30	37	19	1.8	7.1	67
E1154K	205	46	129	13	4.6	7.6	64
F1174L	199	57	484	19	5.7	53	227
L1196P	2140	3112	1798	>3000	1854	338	1061
L1198R	230	207	106	59	7.6	7.2	57
T1211I	32	19	23	4.7	0.4	7.0	66
D1232N	45	20	18	8	1.4	7.0	31
C1235R	191	33	110	50	1.8	8.0	42
C1237Y	1230	330	>10,000	>3000	1051	274	488
E1154K + F1174L	159	59	324	40	5.8	31	100
L1196M + G1202R^a^	2401	1429	1826	2461	1964	na	na

*na* not available.

^a^Data from ref. ^19^.

The data represent the average of three or more independent experiments.

Patient 4 showed two variants (E1154K and F1174L) at different frequencies, suggesting the presence of two clones with single mutations or a parent F1174L clone and a derived double-mutant subclone, post-lorlatinib (Table 1). This could not be solved because of limited cfDNA fragments length. F1174 mutants confer resistance to crizotinib and ceritinib, and have been detected in post-lorlatinib samples^7^. However, F1174L alone should not be resistant to lorlatinib^10^. The E1154K variant was previously reported in patients after TKI failure, but was found to confer no resistance^11,12^. The two mutations induced a modest IC_50_ shift in Ba/F3 cells, even when expressed *in cis*, indicating a minor loss of lorlatinib activity (Fig. 2d, e and Supplementary Fig. 3). Therefore, it is unlikely that these mutants alone caused the relapse. When the patient continued lorlatinib beyond progression, the E1154K variant disappeared and F1174L decreased (Table 1), suggesting they were only partially contributing to progression. We cannot exclude that the two mutations were selected by earlier TKIs and persisted on lorlatinib.

Patient 6 showed two mutant clones with substitution of two αE-helix cysteines, C1235R and C1237Y. The two mutations were in trans, as they never appeared on the same reads, thus representing different clones. In Ba/F3 cells, C1235R did not cause any difference in sensitivity to brigatinib, thus questioning its role (Supplementary Fig. 4a) although it did show moderate resistance to other TKIs (Table 2 and Supplementary Fig. 1). By contrast, the C1237Y variant conferred resistance not only to brigatinib (Fig. 2f–g) but also to all tested TKIs (Table 2, Supplementary Fig. 1 and Supplementary Fig. 4b). The patient was subsequently shifted to lorlatinib obtaining a transient stabilization. C1237 mutants resistant to ensartinib were previously observed in vitro^13^. To our knowledge, a mutation in this residue has never been described in patients.

An unusual gatekeeper mutant, L1196P, was identified in patient 14 post-lorlatinib. The mutation was not detected at previous progression on ceritinib. This variant is associated to neuroblastoma in the ClinVar database (Table 1) and is a predicted strong driver by the BoostDM tool^14^, however no information is available regarding its effects on drug sensitivity. The mutation was assayed in Ba/F3 cells and proved to be highly resistant to lorlatinib (Fig. 2h, i), as well as to all other inhibitors (Supplementary Fig. 5a, Table 2 and Supplementary Fig. 1). In addition, Ba/F3 cells expressing the L1196P mutant showed higher basal EML4::ALK autophosphorylation and increased growth rate compared to the WT (Supplementary Fig. 5b, c). Other mutations were detected in patients 10 (D1232N) and 11 (T1211I) but had no effect in Ba/F3 cells (Supplementary Fig. 6a, b). The significance of these two substitutions remains obscure. It is possible that these non-resistant variants conferred a subtle advantage to a clone that acquired additional, ALK-independent, mechanisms of resistance, as previously observed in ALK+ lymphoma^15^.

As C1237Y and L1196P mutants appeared to resist all clinical inhibitors, we tested their sensitivity to two novel investigational drugs, zotizalkib (TPX-0131)^16^ and repotrectinib (TPX-0005)^17^ and compared the compounds activity with their close structural analogue, lorlatinib. While both mutants were highly resistant to lorlatinib (Fig. 2j–k), zotizalkib and repotrectinib showed inhibition of EML4::ALK autophosphorylation and cell proliferation, although only at doses >100 nM, indicating a significant loss of activity (Fig. 2l–o). These results confirm that these two mutations confer broad resistance to TKIs. Zotizalkib and repotrectinib were also profiled against all other mutations: surprisingly, F1174L showed moderate resistance to these drugs (Table 2, Supplementary Fig. 1 and Supplementary Fig. 7).

Molecular modelling was run to elucidate the mechanisms of resistance (Fig. 3a). Leucine 1198 is located at the kinase hinge region between H-bonding residues E1197 and M1199^18^. While a phenylalanine substitution increases affinity for crizotinib^19^, arginine is likely to repulse the charged piperidine moiety of the compound, weakening hinge binding. Molecular dynamics (MD) optimization evidenced how the presence of R1198 moves the piperidine group away, shifting crizotinib outward (Fig. 3b). Cysteine 1237 belongs to the C-lobe αE-helix (Fig. 3a), outside the active site. The αE-helix is involved in allosteric regulation of kinase activity, by controlling αC-helix movement, activation loop positioning, DFG conformation and hydrophobic spine alignment^20–23^. Cysteine 1237 is highly conserved across vertebrates, suggesting an important structural role. We performed MD studies on the ALK-ADP complex and the corresponding C1237Y model in their active conformation. The ADP-C1237Y complex showed a much smaller root-mean-square deviation than ADP-WT, indicating a higher affinity/activity of the mutant (Fig. 3c). Indeed, EML4::ALK^C1237Y^ consistently showed higher basal autophosphorylation compared to WT protein in cells, and Ba/F3 cells expressing the C1237Y mutant showed accelerated growth rate compared to the wild type (Supplementary Fig. 8). Stabilization of the C1237Y mutant enzyme in its active conformation is linked to the formation of a network of H-bonds between Y1237, neighbouring H1247, and the backbone of DFG’s D1270, which locks in place the DFG motif (Fig. 3d). If our hypothesis is correct, the C1237Y mutant has much higher affinity for its natural ligand (ATP) and this would be a great disadvantage for any ATP-competitive inhibitor. These results may explain the general loss of activity of all tested inhibitors against the C1237Y mutant, as the compounds would have decreased binding capacity despite not having a reduced fit into the pocket. Further experimental validation will be important to support this conclusion. Finally, leucine 1196 represents the well-known gatekeeper residue associated with drug resistance^24,25^. The classical crizotinib-refractory L1196M mutant is sensitive to second-generation drugs, as their binding is not affected by the bulkier methionine. However, proline is smaller than leucine and confers stiffness to the hinge: while L1196 interacts hydrophobically with a methyl group of lorlatinib, stabilizing drug binding, P1196 is predicted to lose such interaction (Fig. 3e), while ATP binding is not affected (Supplementary Fig. 9).

**Fig. 3:**
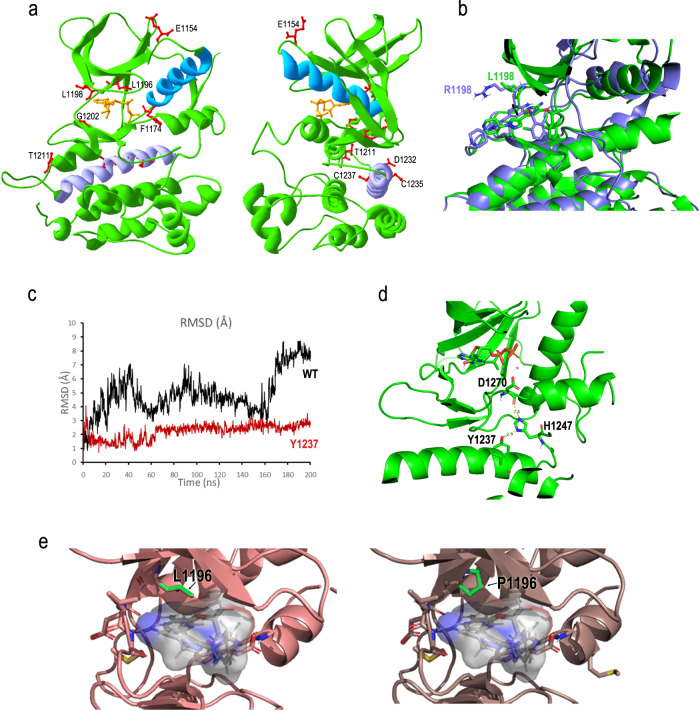
Molecular modelling of ALK mutants. **a** Rotated views of ALK KD in complex with ADP (PDB: 3LCT) showing the position of the residues found mutated in this study (side chains shown as red sticks). The backbone is represented in green ribbon, except: αC-helix in cyan; αE-helix in lilac. ADP is in orange. **b** Superposition between ALK kinase domain (KD) in complex with crizotinib (PDB: 2XP2) in green and the model of R1198 after a 5 ns MD run. **c** RMSD values of the protein-ligand complexes for ALK kinase domain (KD) in complex with ADP (PDB: 3LCT; black line) and the corresponding C1237Y mutant (red). **d** Conformation of the ADP-bound C1237Y mutant highlighting the H bond network across Y1237-H1247-D1270 residues. **e** Close view of lorlatinib (shown as filled space) binding within WT (L1196; *left*) and mutant (P1196; *right*) ALK KD. The mutation disrupts a hydrophobic interaction of lorlatinib with the hinge region.

Plasma genotyping can identify novel TKI-resistant variants that may go undetected with traditional single-site tissue biopsy. We present here new mutated versions of EML4::ALK fusion kinase identified in NSCLC patients relapsing after TKI therapy. Variants were characterized against the whole panel of clinical ALK inhibitors. These data can inform the clinician on the correct sequencing of drugs following a relapse. Three mutants (L1196P, C1237Y and L1196M/G1202R) showed pan-inhibitor resistance. These highly resistant mutants appeared after multiple lines of TKI therapy. It will be interesting to see whether the increasing upfront use of next-generation drugs will be able, in the future, to prevent the rise of these difficult mutations, or if they will represent a serious challenge. The novel L1196P variant adds to the list of drug-resistant gatekeeper mutants of ALK, further confirming the crucial position of this residue inside the enzyme active site. Interestingly, it shows that not only bulky variants, but also smaller amino acids can affect drug binding. The L1198R mutant is of particular interest, considering that different substitutions at L1198 have been described previously with opposite behaviour: while a L1198P mutant was resistant to crizotinib^26^, the L1198F variant is highly sensitive to crizotinib while conferring resistance to other ALK inhibitors^19,27^. Additional variants have been associated with resistance, in the context of compound mutants^28,29^, indicating that L1198 is an important determinant of inhibitor binding.

The presence of some rare mutations, like C1237Y and L1196P, is a relevant finding due to their resistance demonstrated even after the exposure to the newest fourth-generation inhibitor TPX-0131. In clinical practice, second/third generation ALK TKIs are being moved upfront and the clonal selection of these specific mutations may lead to a complete refractory status to these new compounds, which would then be useless in a sequential therapeutic strategy. This study has limitations: the small sample size, the lack of comparison with tissue biopsies at relapse, missing liquid biopsies at earlier timepoints, and the lack of information on by-pass mechanisms in ALK WT patients. A larger, longitudinal study focusing on a wider gene panel is currently underway to capture a more comprehensive description of TKI resistance mechanisms in these patients.

## Methods

### Patients and study design

Liquid biopsies from consecutive patients treated at the Fondazione IRCCS San Gerardo dei Tintori, Monza, Italy were collected. Patients were enrolled based on the following eligibility criteria: minimum 18 years of age; histological diagnosis of locally advanced or metastatic lung cancer; positivity for genetic rearrangements of ALK or ROS1, as identified on tissue samples by a clinically validated method (immunohistochemistry, FISH or NGS); radiological progression according to RECIST 1.1 criteria to treatment with specific ALK/ROS1 inhibitors in any treatment line. Patients pre-treated with chemotherapy, as well as patients with active brain metastases, were eligible. The study was approved by the local ethical committee (Comitato Etico Brianza) and conducted in accordance with the Declaration of Helsinki (ClinicalTrials.gov Identifier: NCT06081270). Written informed consent was obtained from every participant. The primary endpoint was the identification of novel mutations in ALK and ROS1 genes, which determined resistance to TKI treatment. Progression-free survival is defined as the period from the first day of each TKI treatment until the date of progression or death from any cause and overall survival (OS) is defined as the time interval between the date of diagnosis and the date of death from any cause. Survival curves were estimated by Kaplan-Meier method and compared by log-rank test.

### Extraction of cfDNA

Ten to fifteen millilitres of peripheral blood were collected in K2-EDTA tubes and processed within 2 hours. Plasma was obtained from whole blood by double centrifugation, as described^30^ and stored at −80 °C until used. Circulating free DNA (cfDNA) was isolated from plasma using the QIAmp MinElute ccfDNA Mini Kit (Qiagen) according to the manufacturer’s instructions. Extracted cfDNA was quantified using Qubit DNA HS Assay (Thermo Fisher) and by estimation of amplifiable DNA by qPCR with Alu primer pairs^31^. Fragment size was determined with High Sensitivity D5000 ScreenTape (Agilent). Median DNA yield was 4.8 ng (interquartile range [IQR], 2.9–8.3 ng) per ml of plasma and median fragment size was 176 bp (IQR 169–185).

### Next-generation sequencing and bioinformatic analysis

ALK exons 22 to 25, and ROS1 exons 37, 38, and 41, covering major mutational hotspots of the two genes, were amplified by high-fidelity PCR starting from purified cfDNA, using specific primers (Supplementary Table 2). Amplicons were purified from agarose gel using the QIAquick Gel Extraction kit (Qiagen), quantified by Qubit assay and sent to Galseq srl (Bresso, Italy) for library preparation and sequencing. Median coverage of called variants was 24,687x (IQR 12,988–64,874). Fastq raw data were aligned on the hg38 human reference genome using BWA-MEM (with default settings) and Bowtie2 (with ‘very sensitive local’ preset parameter settings) aligners. Analysis of background signal on healthy control DNA identified 0.0024 mismatches per mapped base (Supplementary Fig. 10). Hence, the threshold was set at 0.6% for mutation calling. Variants were called using LoFreq^32^ and posteriorly filtered with LoFreq Filter tool, using the following settings: minimum baseQ=6, call quality and strand bias filtering FDR < 0.001, minimum coverage 5000x, gnomAD allele fraction <10^−5^, minimum alternative allele frequency 0.6%. Variants passing filters with both alignment tools were considered ‘strong evidence’, while those detected by only one of the two methods were labelled as ‘weak evidence’ calls and were discarded. All mutations were manually inspected using IGV software.

### Reporting summary

Further information on research design is available in the Nature Research Reporting Summary linked to this article.

### Supplementary information

Supplementary data

REPORTING SUMMARY

## Data Availability

The data that support the findings of this study are available from the corresponding author upon reasonable request. Sequencing fastq files were deposited in the Sequence Read Archive (BioProject ID: PRJNA1053258).
