# Supplementary material for: New pan-ALK inhibitor-resistant EML4::ALK mutations detected by liquid biopsy in lung cancer patients
Source: NPJ Precis Oncol. 2024 Mar 6;8:29. doi: 10.1038/s41698-024-00498-w (PMC10918084; doi:10.1038/s41698-024-00498-w)
Supplement: Supplementary file 1 — Supplementary data [file 41698_2024_498_MOESM1_ESM.docx]

**SUPPLEMENTARY DATA**

**Supplementary Methods**

Site-directed mutagenesis and generation of Ba/F3 cell models

The pCDH-CMV-MCS-EF1-Puro vector containing wild-type EML4::ALK (a kind gift from Dr. Robert Doebele, University of Colorado) was mutagenized as previously described ^1^ to obtain the desired variants, using oligonucleotides reported in **Supplementary Table 3**. All mutations were confirmed by Sanger sequencing. The murine IL3-dependent pro-B-cell line Ba/F3 was maintained in RPMI 1640 supplemented with 10% fetal bovine serum, 2 mM L-glutamine, 100 U/mL penicillin and 100 μg/mL streptomycin (Euroclone). Ba/F3 cells were stably transfected with wild-type or mutated pCDH-EML4::ALK plasmid by nucleofection, using the SG Cell Line 4D-Nucleofector X Kit (Lonza) as recommended by the manufacturer, and selection with puromycin (1 μg/mL). ALK-transformed Ba/F3 clones were selected by IL3 withdrawal and tested for EML4::ALK expression by Western blot.

Cell viability assays and Western blotting

Compounds were purchased from Selleck Chem, dissolved in DMSO, aliquoted and stored at -20˚C. Cell viability was assessed by colorimetric MTS assay (CellTiter 96® AQueous One Solution Cell Proliferation Assay, Promega). Cells (10,000/well) were seeded in 96-well plates and treated with semi-log increasing concentrations of inhibitors, or DMSO (vehicle). After 72 hours, the MTS reagent was added to the wells and absorbance was read after 1-3 hours using a TECAN Infinite F200 Pro plate reader. Dose-response curves were built using GraphPad Prism software, by non-linear regression of the data using the log(inhibitor) *vs* normalized response fitting. The IC_50_ value was calculated as the 50% inhibitory drug concentration. For Western blotting, the cells were seeded in 6-well plates and treated with inhibitors for 4 hours. Equal amounts of total cell lysates were run on SDS-PAGE and analyzed as described,^2^ using phospho-ALK (Tyr1604) and total ALK (31F12) antibodies from Cell Signaling Technology. Uncropped blot images are shown as **Supplementary figures** **11-17**. All blots derive from the same experiment and were processed in parallel.

Molecular modelling

Modelling studies were performed using the Maestro suite from Schrodinger (Maestro version 13.4.134, MMshare Version 6.0.134, Release 2022-4). We obtained the crystal structure of ALK in complex with lorlatinib (PDB ID: 4CLI), crizotinib (PDB ID: 2XP2) and ADP (PDB ID: 3LCT) in its active conformation. The structures were prepared using the Protein Preparation Workflow routine, after which models of the L1196P, L1198R and C1237Y mutants for all pdb entries were produced and the resulting structures were subjected to short minimization. Molecular dynamics simulations (200 ns) were carried out on the ADP-bound C1237Y model and the corresponding native protein-ADP complex to investigate the relative stabilities of the two conformations, while shorter (5 ns) simulations were run on the crizotinib-bound L1198R model and the corresponding WT complex to evaluate the effect of the mutation on crizotinib binding. The structures were solvated in a triclinic box using the simple point charge (SPC) water model, Na^+^ and Cl^–^ ions corresponding to 0.15 M concentration were added as well as Na^+^ counterions to neutralize the system. The final simulation box measured 100 Å along each side. The OPLS4 force field72 and Desmond MD system (Schrödinger Release 2022-4) as implemented within Schrödinger Suite (release 2022-4) were used in this study. The systems were initially minimized and equilibrated with restraints on all solute heavy atoms, followed by production runs. The constant-temperature, constant-pressure NPT ensemble was used with constant temperature at 300 K and Langevin dynamics. The runs on the ADP-bound C1237Y model and the corresponding native protein-ADP complex were carried out for 200 ns and the trajectories were analyzed with the Simulation Interactions Diagram routine of Maestro.

**Supplementary Table 1.** PFS, lines of therapy, response and target gene sequence of the 20 samples analyzed (excluding the one TBP sample from patient 4). Samples taken at second time point from the same patient are indicated by the letter *B*. Therapy line includes chemotherapy.

PR=partial response; SD=stable disease; PD=progressive disease.

| **ID** | **INHIBITOR** | **PFS (months)** | **THERAPY**  **LINE** | **TKI**  **LINE** | **BEST RESPONSE** | **SEQUENCE** |
| --- | --- | --- | --- | --- | --- | --- |
| **1** | brigatinib | 10 | 2 | 2 | PR | **mut** |
| **2** | crizotinib | 14 | 1 | 1 | PR | **mut** |
| **3** | crizotinib | 21 | 2 | 1 | PR | wt |
| **3B** | alectinib | 15 | 3 | 2 | SD | wt |
| **4** | lorlatinib | 8 | 4 | 4 | SD | **mut** |
| **5** | crizotinib | 7 | 2 | 1 | SD | wt |
| **6** | brigatinib | 16 | 3 | 2 | SD | **mut** |
| **7** | crizotinib | 26 | 1 | 1 | SD | wt |
| **8** | alectinib | 28 | 3 | 2 | SD | wt |
| **9** | lorlatinib | 25 | 3 | 2 | PR | wt |
| **10** | alectinib | 45 | 4 | 2 | PR | **mut** |
| **11** | alectinib | 2 | 1 | 1 | PD | **mut** |
| **12** | crizotinib | 20 | 1 | 1 | PR | wt |
| **13** | alectinib | 13 | 2 | 1 | SD | wt |
| **13B** | lorlatinib | 14 | 3 | 2 | PR | wt |
| **14** | ceritinib | 78 | 2 | 1 | PR | wt |
| **14B** | lorlatinib | 4 | 3 | 2 | SD | **mut** |
| **15** | crizotinib | 16 | 1 | 1 | SD | wt |

**Supplementary Table 2.** Primers used in this study to amplify ALK and ROS1 exons.

| Gene | Exon No. | Forward  (5’-3’) | Reverse  (5’-3’) | Amplicon size |
| --- | --- | --- | --- | --- |
| ALK | 22 | CACCCTCCCCTTCTCTGC | GGGTGTCTCTCTGTGGCTTT | 109 |
| ALK | 23 (1) | TACATCCCTCTCTGCTCTGC | AGGGATTGCAGGCTCACC | 76 |
| ALK | 23 (2) | GGTGAGCCTGCAATCCCT | CAGCAAAGACTGGTTCTCACTC | 118 |
| ALK | 24 | CGCTTCTGTCTCCCCACA | ATCCACCGGTGAGTCAAAGT | 129 |
| ALK | 25 | CTTCCCAGAGACATTGCTGC | GTAAAGACTGCCTCACCCCT | 125 |
| ROS1 | 37 | GTCTGCTATACTGATTCCTGACTTGT | AAACTTCTTACCCATACCAGGAGA | 148 |
| ROS1 | 38 | GCCTGTGTCTTTCCACCTTTCA | TCCCAACTGCCTACCGTTGC | 167 |
| ROS1 | 41 | GCAAACCCTCTACTATTCTTAGGTC | CAGATCATCAGGACAATTTCTTGG | 158 |

**Supplementary Table 3.** Oligonucleotides for site-directed mutagenesis. The mutated nucleotides are shown in red. Only the sense oligo is shown, the antisense being its reverse complementary.

| **Mutation** | **Sequence (sense oligo, 5’-3’)** |
| --- | --- |
| **E1154K** | CTGTGAAGACGCTGCCTAAAGTGTGCTCTGAACAG |
| **F1174L** | GCCCTGATCATCAGCAAACTCAACCACCAGAACATTG |
| **L1196P** | CAATCCCTGCCCCGGTTCATCCTGCCGGAGCTCATGGCGGG |
| **L1198R** | GTTCATCCTGCTGGAGCGCATGGCGGGGGGAGAC |
| **T1211I** | CCTTCCTCCGAGAGATCCGCCCTCGCCCGAG |
| **D1232N** | TCTGCACGTGGCTCGGAACATTGCCTGTGGCTGT |
| **C1235R** | CGTGGCTCGGGACATTGCCCGTGGCTGTCAGTATTTGGAG |
| **C1237Y** | CGGGACATTGCCTGTGGCTATCAGTATTTGGAGGAAAACC |

**SUPPLEMENTARY FIGURE 1**

| **Relative Resistance index** | **crizotinib** | **brigatinib** | **ceritinib** | **alectinib** | **lorlatinib** | **zotizalkib** | **repotrectinib** |
| --- | --- | --- | --- | --- | --- | --- | --- |
| **WT** | 1 | 1 | 1 | 1 | 1 | 1 | 1 |
| **E1154K** | 2.3 | 1.5 | 3.5 | 1 | 2.6 | 1 | 1 |
| **F1174L** | 2.2 | 1.9 | 13 | 1 | 3.2 | 7.5 | 3.4 |
| **L1196P** | 24 | 104 | 49 | 158 | 1030 | 48 | 16 |
| **L1198R** | 2.6 | 6.9 | 2.9 | 3.1 | 4.2 | 1 | 1 |
| **T1211I** | 0.4 | 1 | 1 | 0.2 | 0.2 | 1 | 1 |
| **D1232N** | 0.5 | 1 | 0.5 | 0.4 | 1 | 1 | 0.5 |
| **C1235R** | 2.1 | 1 | 3.0 | 2.6 | 1 | 1 | 1 |
| **C1237Y** | 14 | 11 | 270 | 158 | 584 | 39 | 7.3 |
| **E1154K+F1174L** | 1.8 | 2.0 | 8.8 | 2.1 | 3.2 | 4.4 | 1.5 |
| **L1196M+G1202R** | 27 | 48 | 49 | 130 | 1091 | na | na |

**Supplementary Figure 1.** **Relative Resistance Index (RRI).** We define RRI as the IC_50_ fold increase obtained with mutants *vs* WT Ba/F3-EML4::ALK cells (see Table 2 for the original IC_50_ data). A gradient green-yellow-orange-red color scale increasing incrementally from RRI=1 (green) to RRI>10 (red) is used to facilitate comparisons of sensitive *vs* refractory mutations.

**SUPPLEMENTARY FIGURE 2**


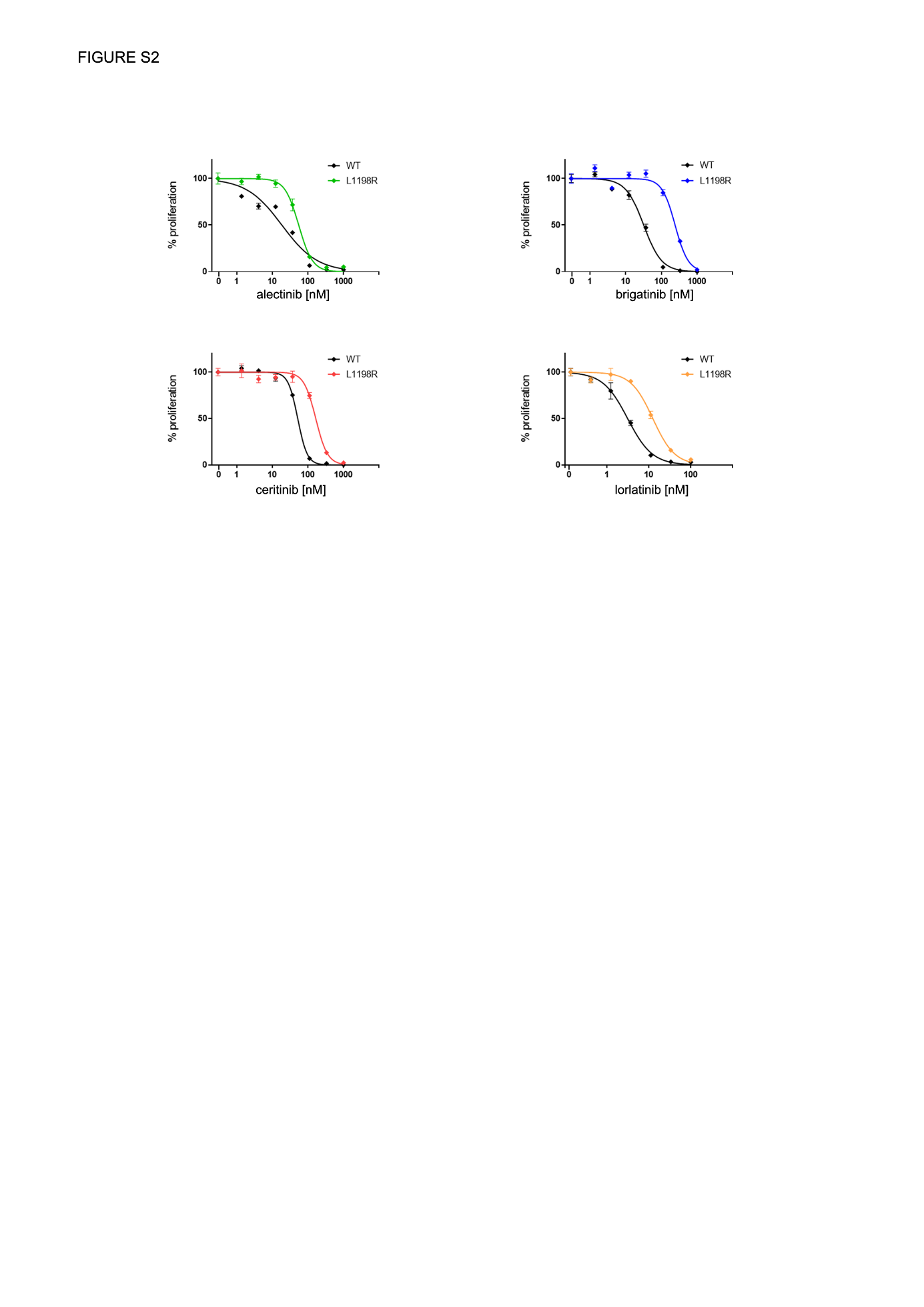
 **Supplementary Figure 2.** Dose-response curves of Ba/F3 cells expressing L1198R (colored lines) and wild-type (WT; black lines) EML4::ALK, treated with the indicated ALK inhibitors.

**SUPPLEMENTARY FIGURE 3**


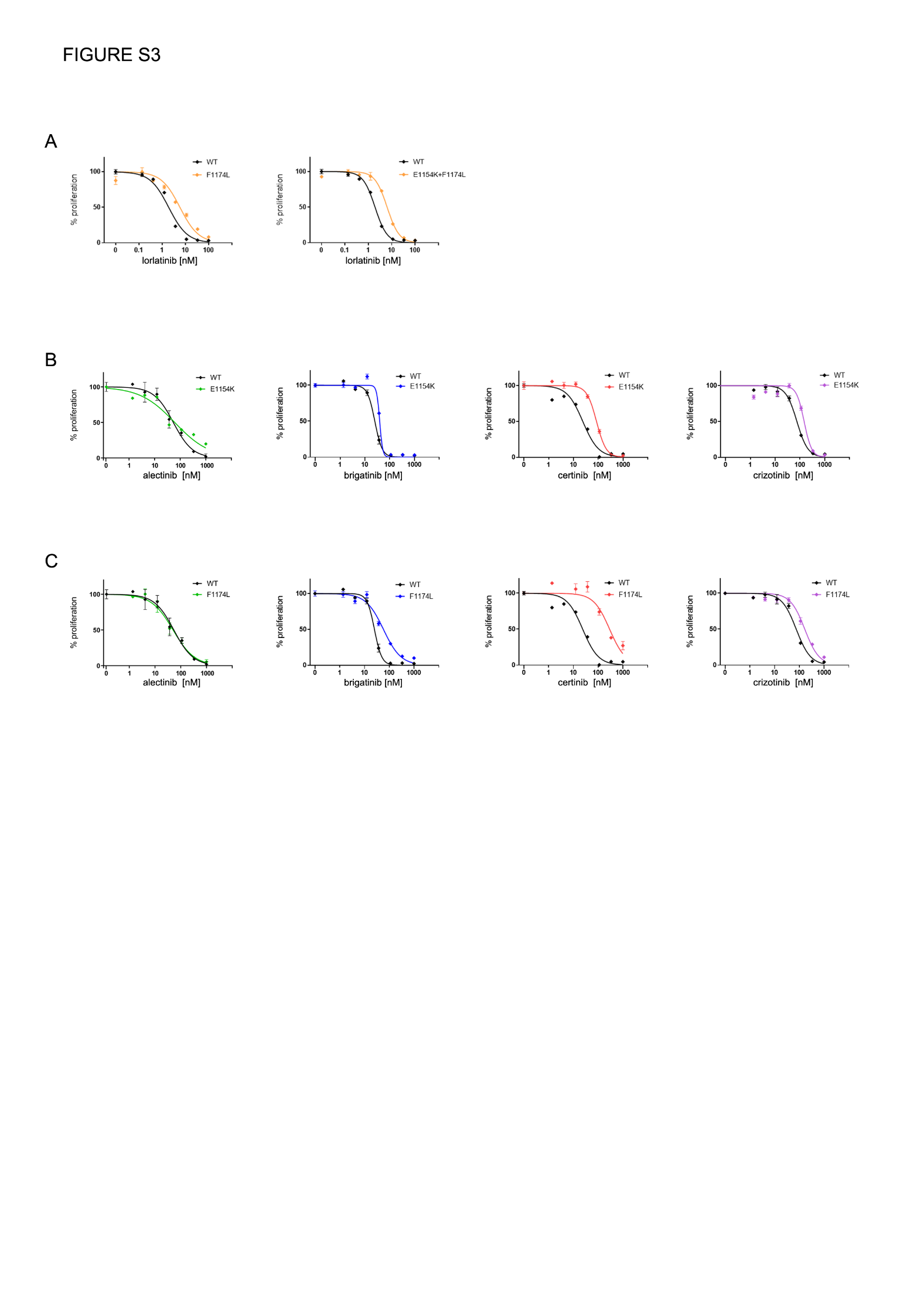


**Supplementary Figure 3.** (**A**) Effect of F1174L and double E1154K/F1174L mutations (orange) on sensitivity to lorlatinib. compared to wild-type EML4::ALK (WT; black lines). (**B-C**) Dose-response curves of Ba/F3 cells expressing E1154K (**B**) or F1174L mutants (**C**) *vs* WT. treated with the indicated ALK inhibitors.

**SUPPLEMENTARY FIGURE 4**


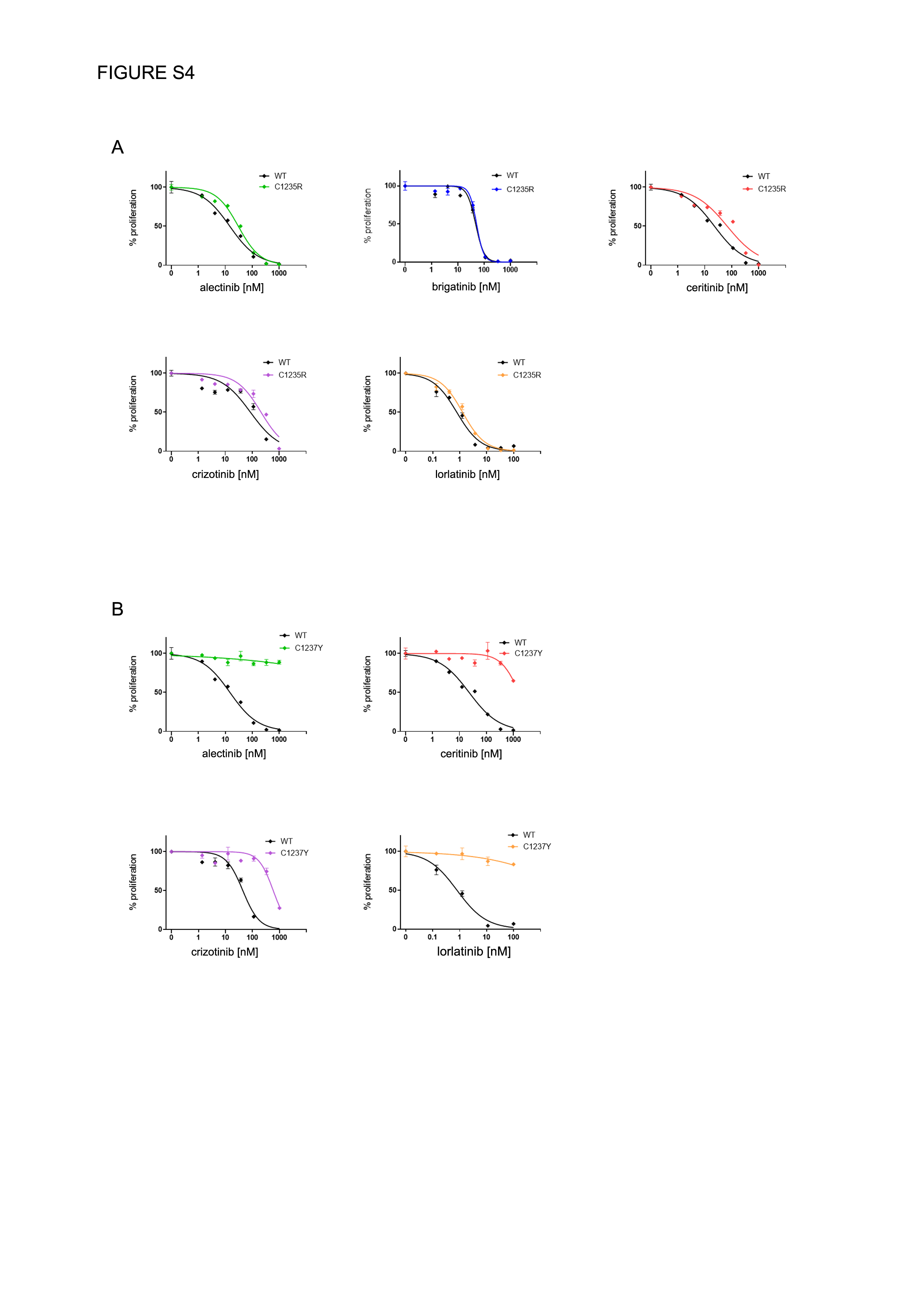


**Supplementary Figure 4.** Dose-response curves of Ba/F3 cells expressing C1235R (**A**) or C1237Y (**B**) mutants (colored lines) compared to WT (black lines). treated with the indicated ALK inhibitors.

**SUPPLEMENTARY FIGURE 5**


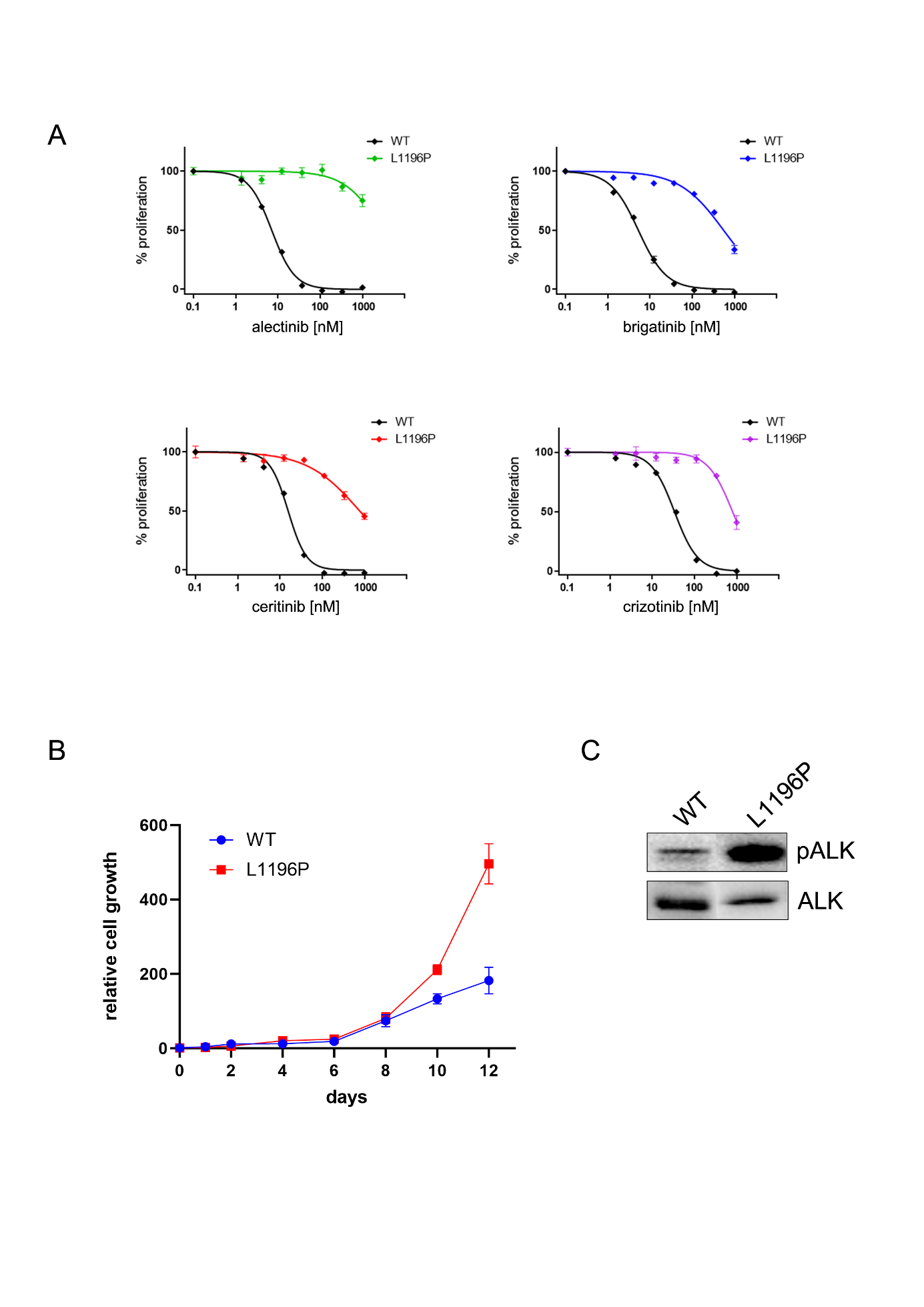


**Supplementary Figure 5.** (**A**) Dose-response curves of Ba/F3 cells expressing L1196P (colored lines) and wild-type (WT; black lines) EML4::ALK. treated with the indicated ALK inhibitors. (**B**) L1196P mutation enhances cell proliferation of Ba/F3-EML4::ALK cells. Time course of cell growth measured by MTS assay. normalized over day 0. is shown. (**C**) Western blotting analysis of EML4::ALK autophosphorylation in Ba/F3 cells expressing wild-type (WT) or L1196P mutant kinase. in the absence of inhibitors. The image is representative of three independent experiments.

**SUPPLEMENTARY FIGURE 6**


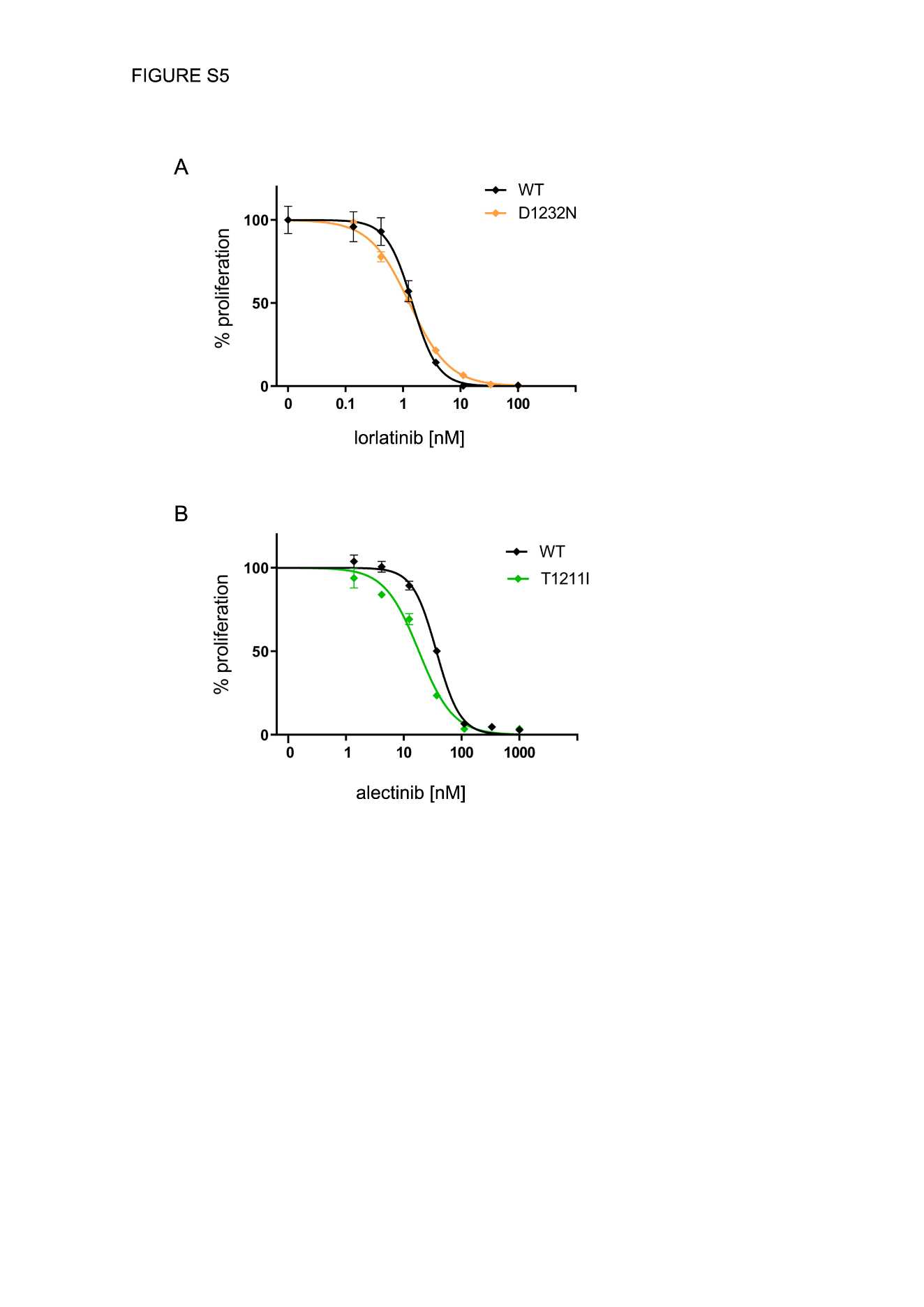


**Supplementary Figure 6.** Dose-response curves of Ba/F3 cells expressing D1232N (**A**) or T1211I (**B**) EML4::ALK. treated with the lorlatinib and alectinib. respectively. Neither mutant confers TKI resistance *in vitro* to the drug used in the corresponding patient.

**SUPPLEMENTARY FIGURE 7**


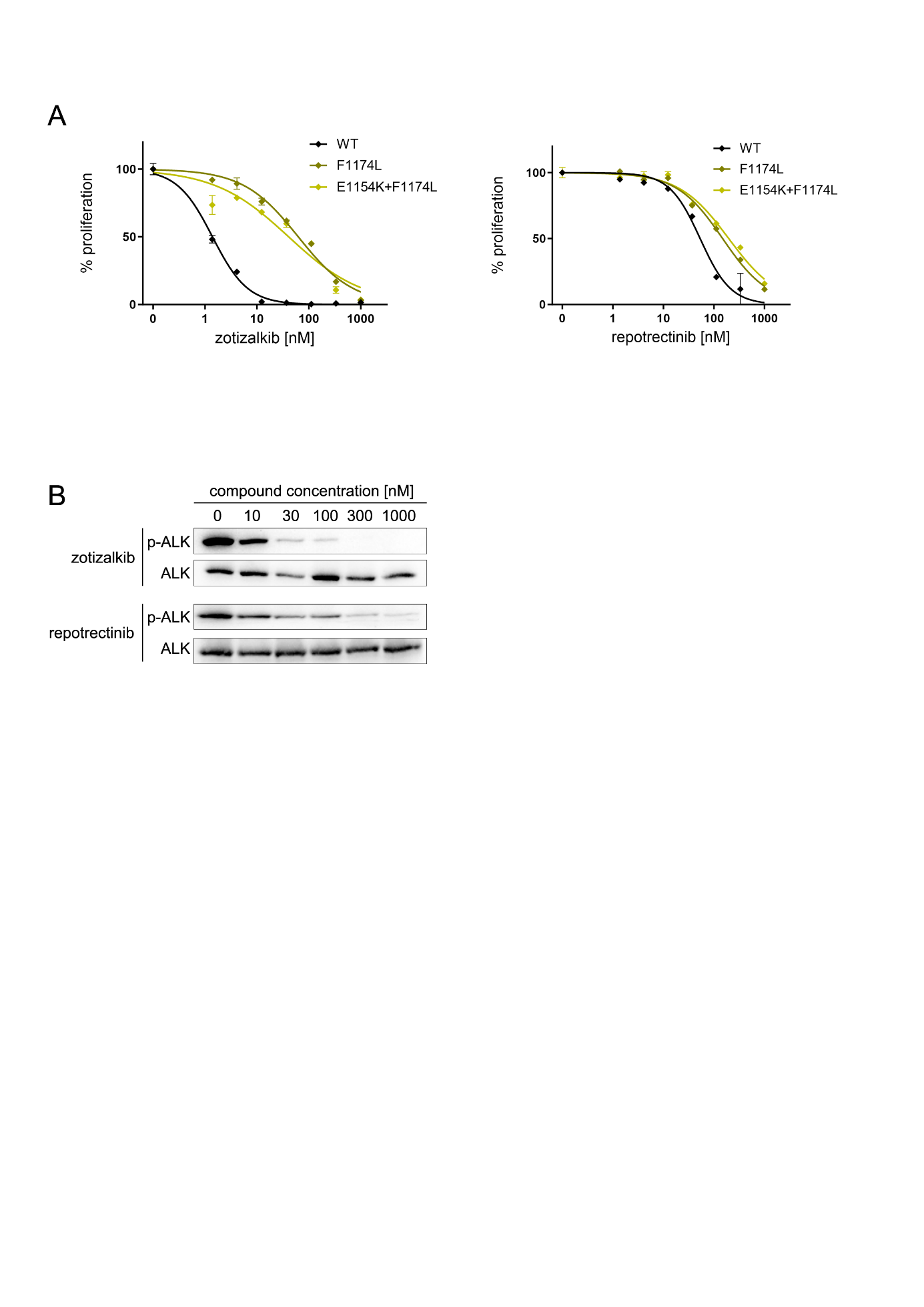


**Supplementary Figure 7.** (**A**) Dose-response curves of Ba/F3 cells expressing F1174L or the compound E1154K+F1174L mutation. treated with zotizalkib (left) and repotrectinib (right). (**B**) Western blot confirmation of moderate resistance of F1174L mutant against zotizalkib and repotrectinib.

**SUPPLEMENTARY FIGURE 8**


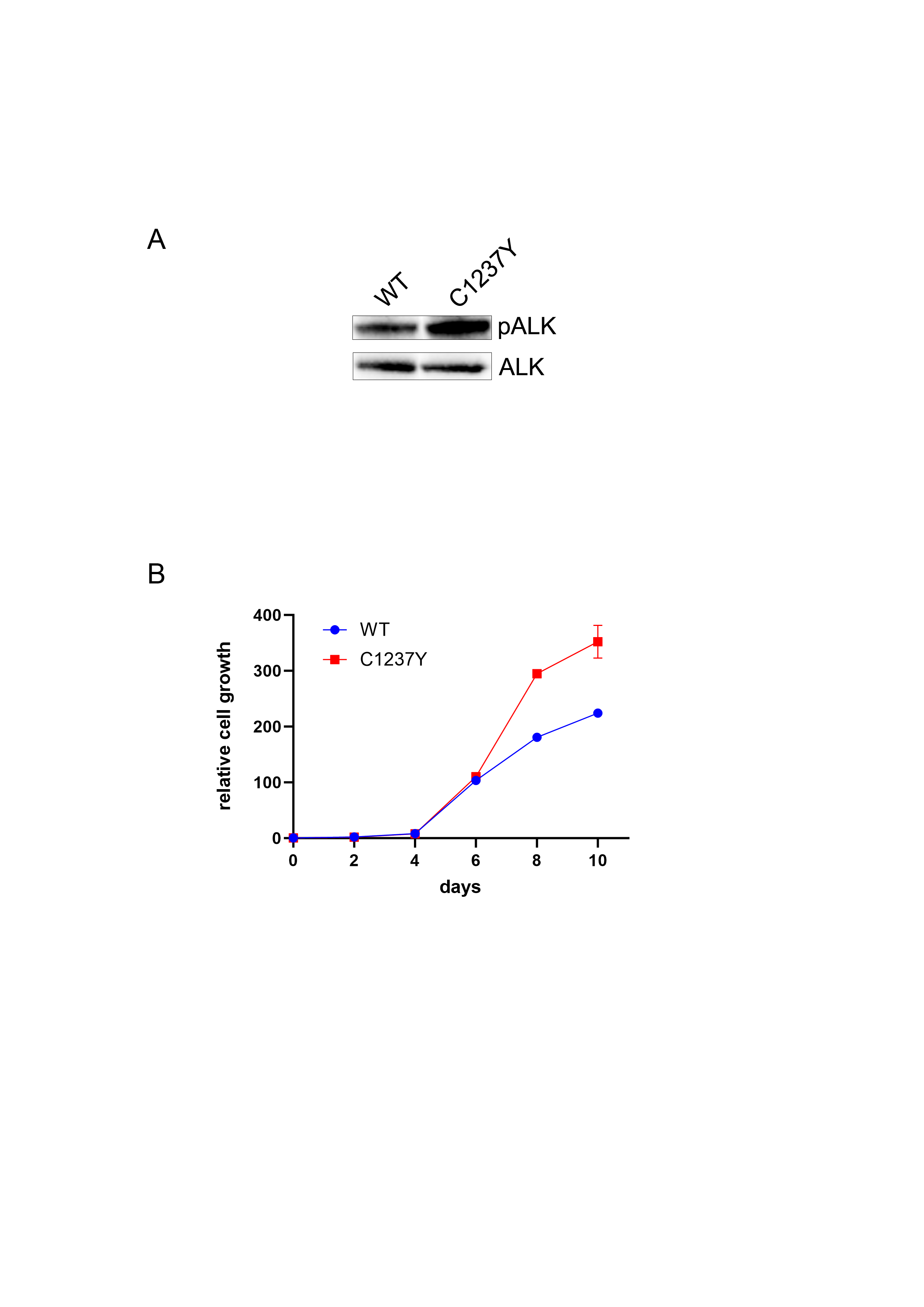


**Supplementary Figure 8.** (**A**) Western blotting analysis of EML4::ALK autophosphorylation in Ba/F3 cells expressing wild-type (WT) or C1237Y mutant kinase. in the absence of inhibitors. The image is representative of three independent experiments. (**B**) C1237Y mutation enhances cell proliferation of Ba/F3-EML4::ALK cells. Time course of cell growth measured by MTS assay. normalized over day 0. is shown.

**SUPPLEMENTARY FIGURE 9**


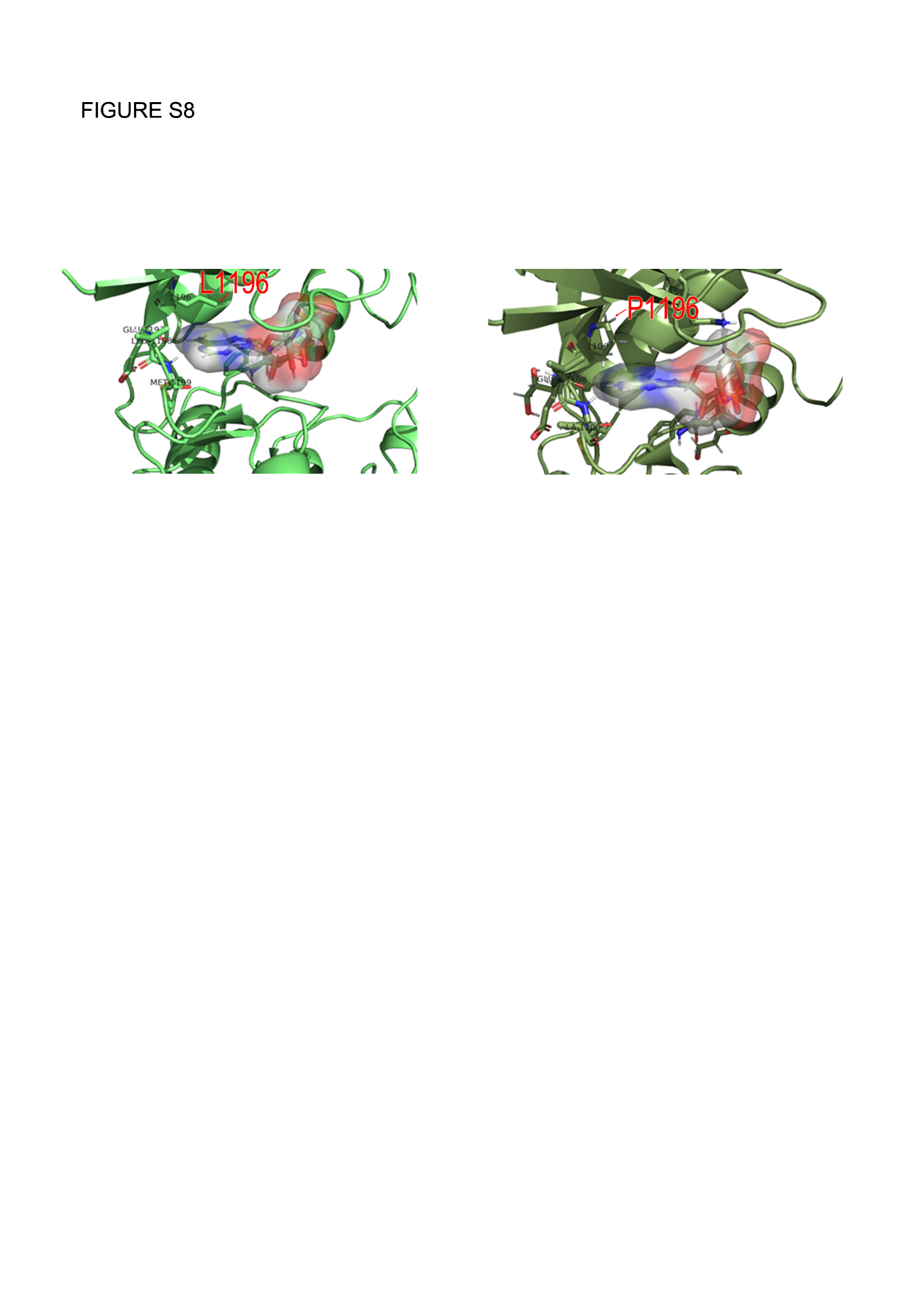


**Supplementary Figure 9.** ATP docking within WT (L1196; *left*) and mutant (P1196; *right*) ALK KD. The mutation does not seem to have any impact on ATP binding.

**SUPPLEMENTARY FIGURE 10**


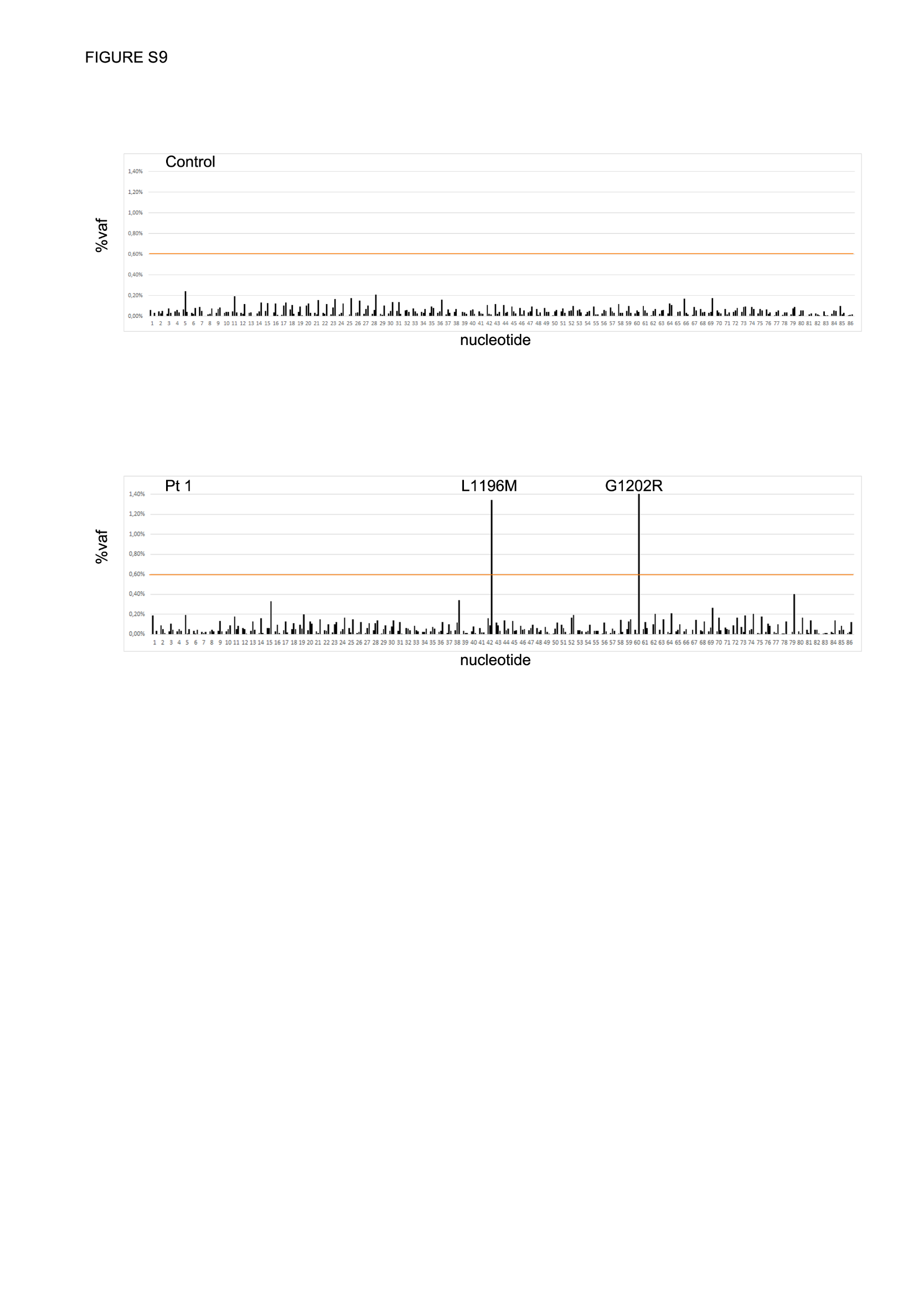


**Supplementary Figure 10.** Alternative allele frequencies across the coding sequence of a representative amplicon (ALK exon 23. 2^nd^ part) taken from control DNA (*upper panel*) and Patient 1 (*lower*). The orange line indicates the threshold (0.6%) for mutation call.

**SUPPLEMENTARY FIGURE 11**

**
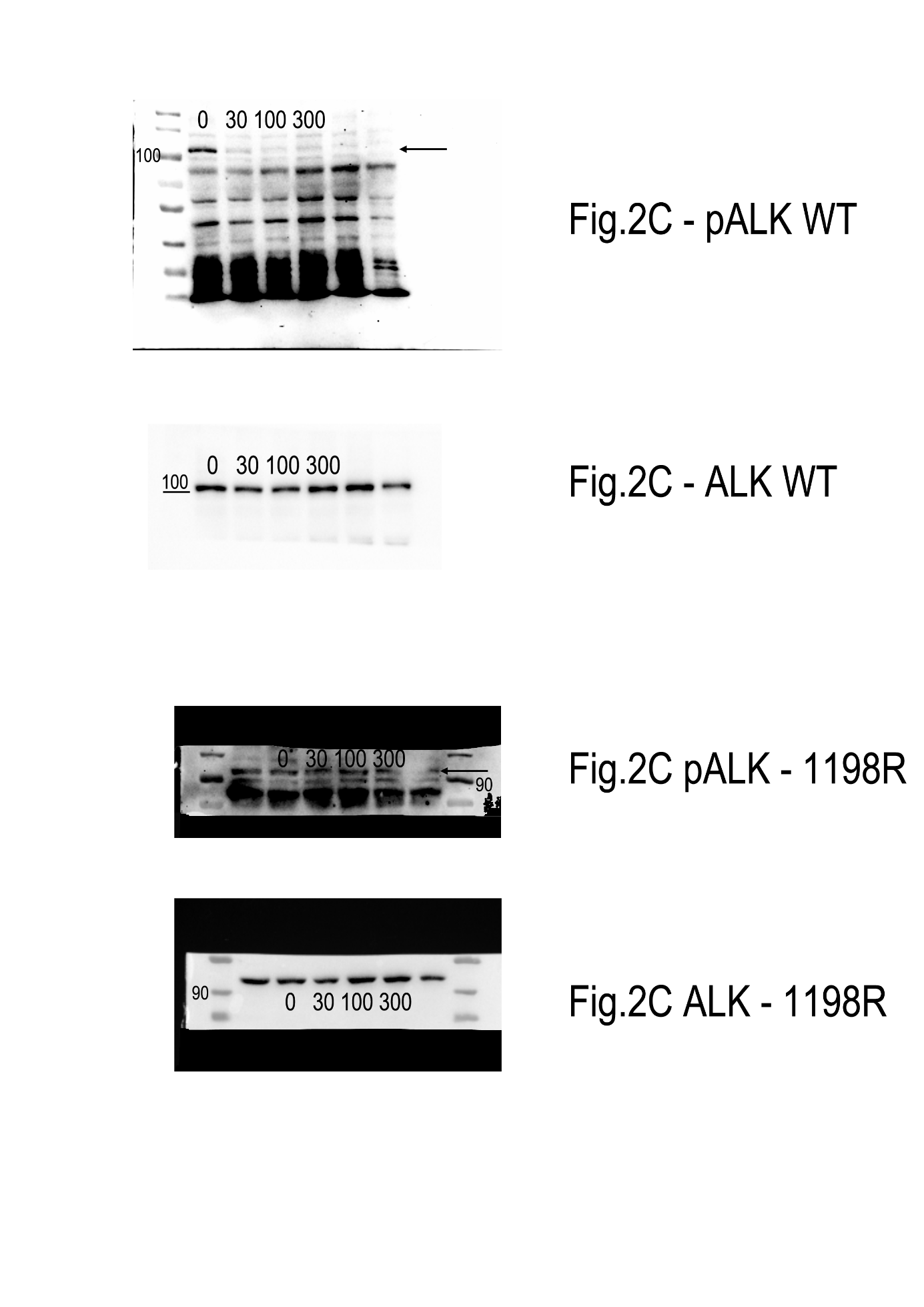
**

**Supplementary Figure 11.** Uncropped mages of Figure 2c.

**SUPPLEMENTARY FIGURE 12**

**
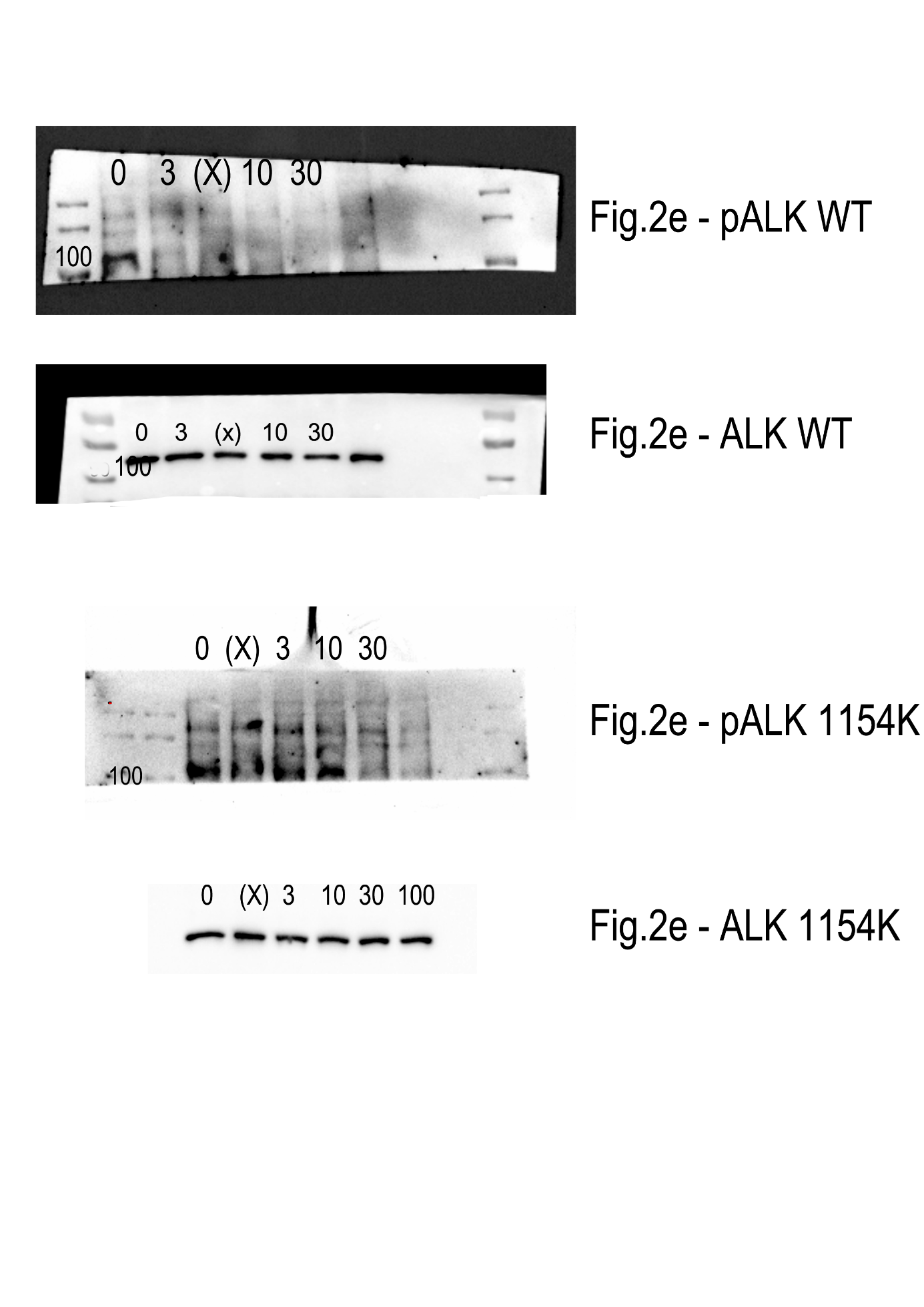
**

**Supplementary Figure 12.** Uncropped mages of Figure 2e.

**SUPPLEMENTARY FIGURE 13**

**
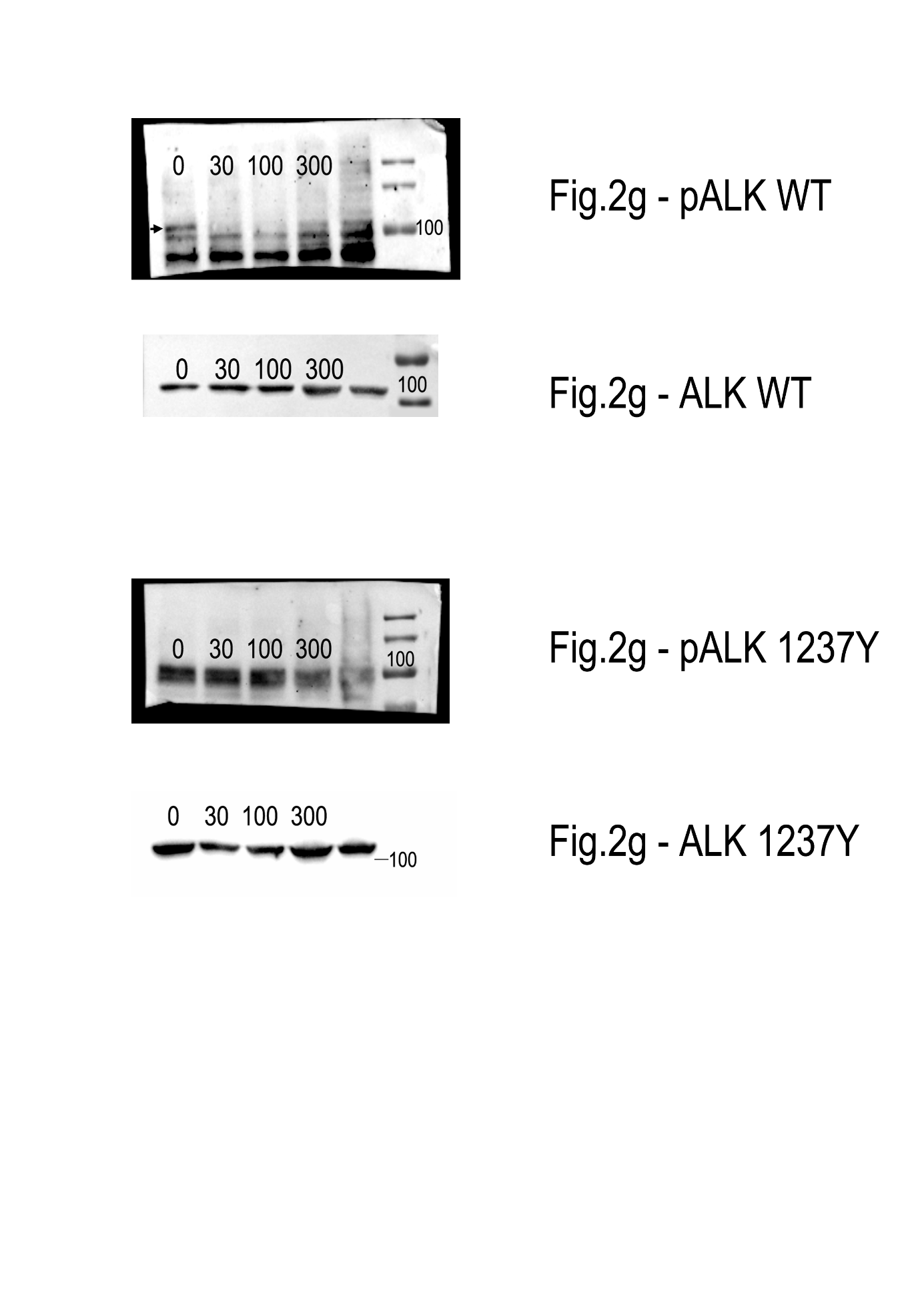
**

**Supplementary Figure 13.** Uncropped mages of Figure 2g.

**SUPPLEMENTARY FIGURE 14**

**
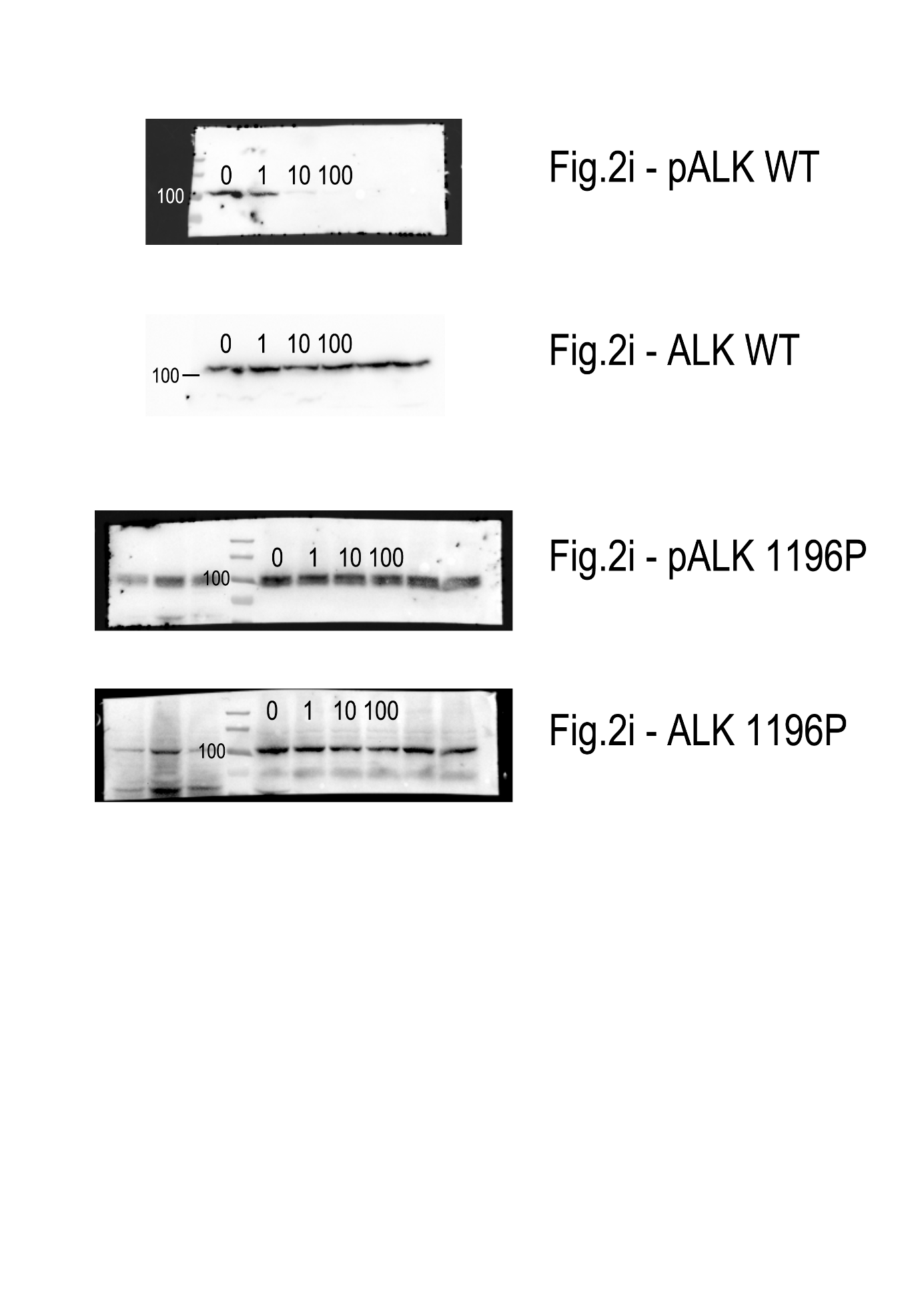
**

**Supplementary Figure 14.** Uncropped mages of Figure 2i.

**SUPPLEMENTARY FIGURE 15**

**
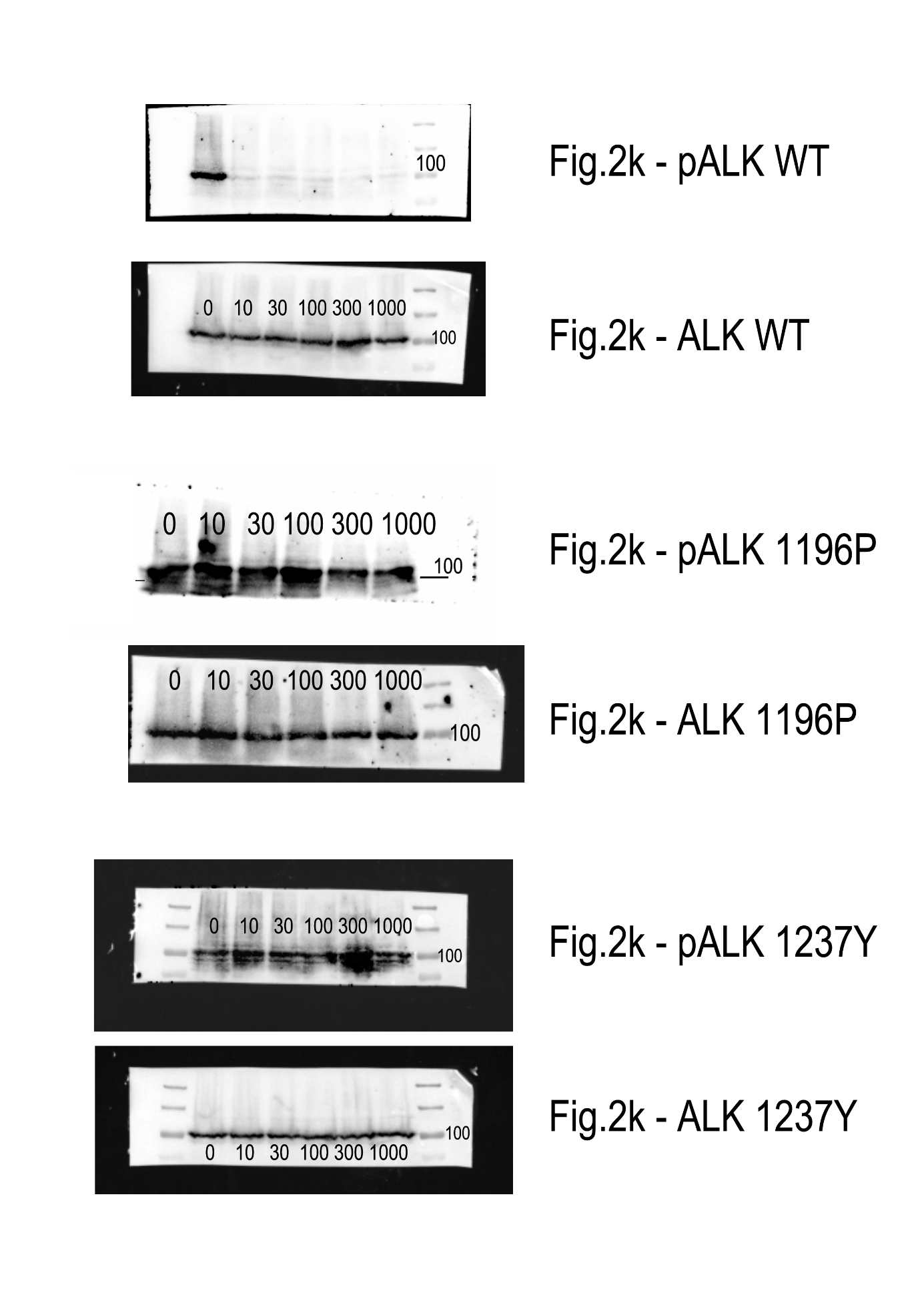
**

**Supplementary Figure 15.** Uncropped mages of Figure 2k.

**SUPPLEMENTARY FIGURE 16**

**
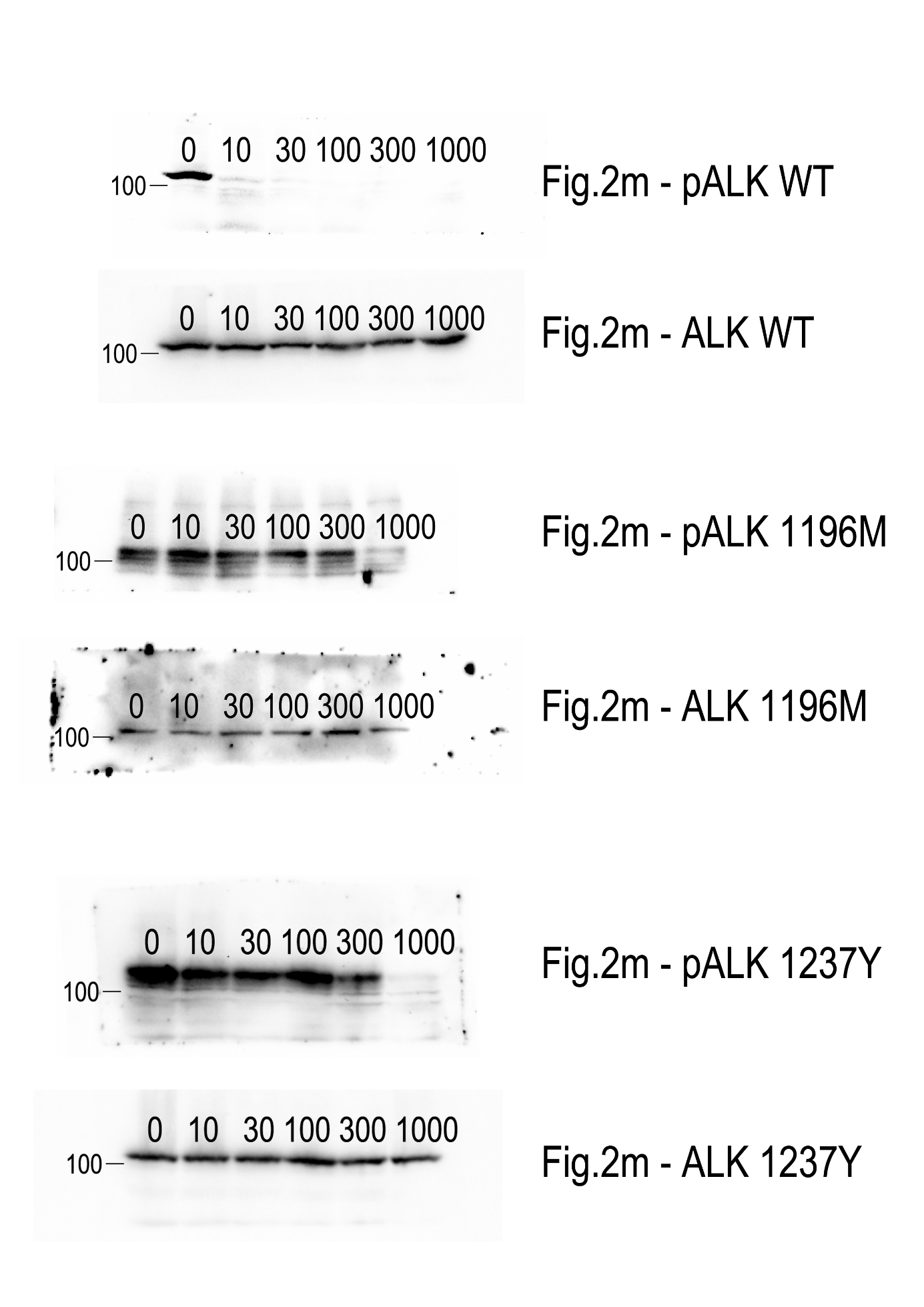
**

**Supplementary Figure 16.** Uncropped mages of Figure 2m.

**SUPPLEMENTARY FIGURE 17**

**
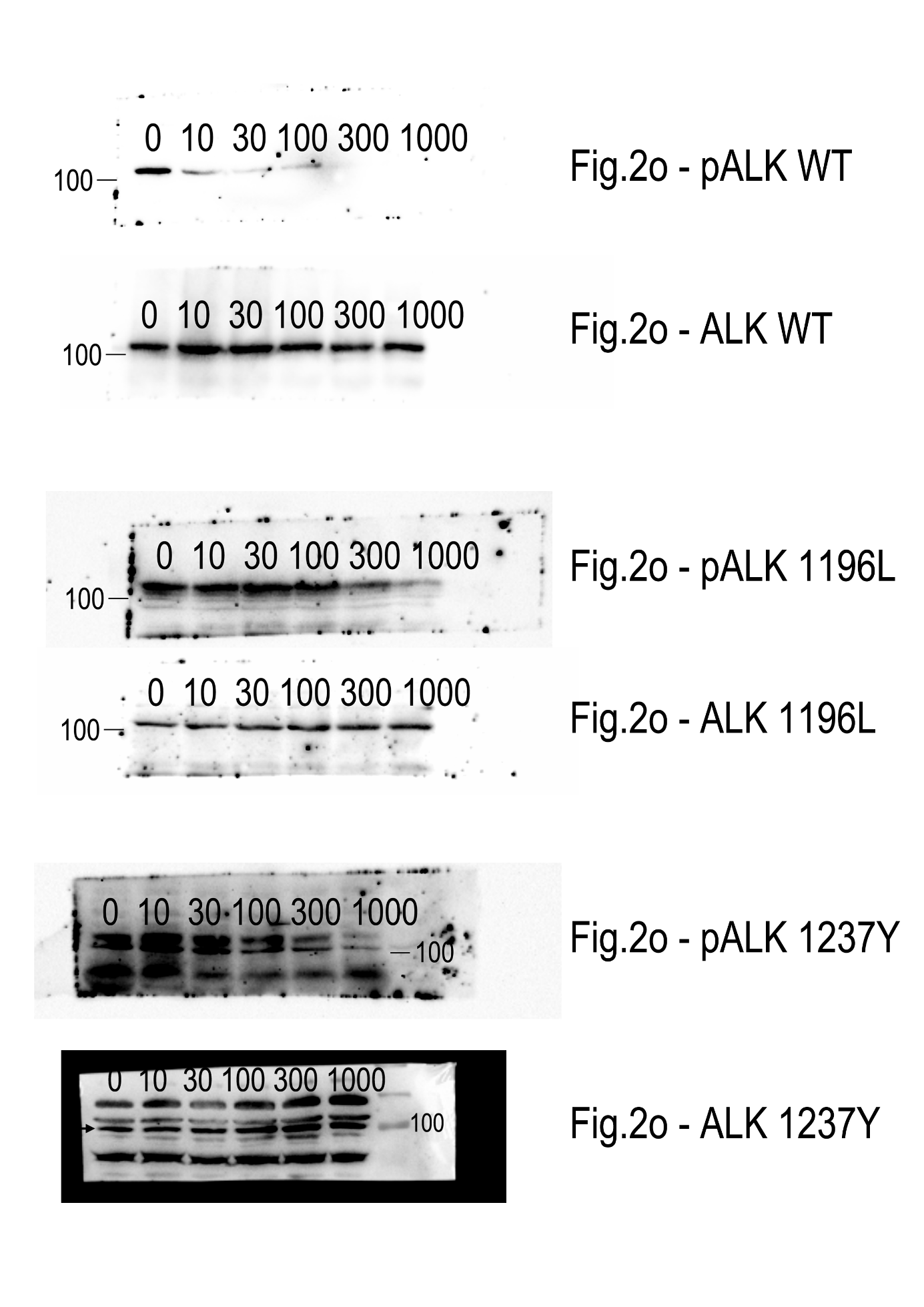
**

**Supplementary Figure 17.** Uncropped mages of Figure 2o.

**REFERENCES**

1. Fontana D. Ceccon M. Gambacorti-Passerini C. Mologni L. Activity of second-generation ALK inhibitors against crizotinib-resistant mutants in an NPM-ALK model compared to EML4-ALK. *Cancer Med.* 2015;4(7):953–65.

2. Arosio G. Sharma GG. Villa M. et al. Synergistic Drug Combinations Prevent Resistance in ALK+ Anaplastic Large Cell Lymphoma. *Cancers (Basel).* 2021;13(17):.
