# Supplementary material for: New pan-ALK inhibitor-resistant EML4::ALK mutations detected by liquid biopsy in lung cancer patients
Source: NPJ Precis Oncol. 2024 Mar 6;8:29. doi: 10.1038/s41698-024-00498-w (PMC10918084; doi:10.1038/s41698-024-00498-w)
Supplement: Supplementary file 2 — REPORTING SUMMARY [file 41698_2024_498_MOESM2_ESM.pdf]

nature portfolio

Corresponding author(s): Luca Mologni

Last updated by author(s): Nov 15, 2023

Reporting Summary

Nature Portfolio wishes to improve the reproducibility of the work that we publish. This form provides structure for consistency and transparency in reporting. For further information on Nature Portfolio policies, see our Editorial Policies and the Editorial Policy Checklist.

Statistics

For all statistical analyses, confirm that the following items are present in the figure legend, table legend, main text, or Methods section.

n/a	Confirmed	
☒	☐	The exact sample size (*n*) for each experimental group/condition, given as a discrete number and unit of measurement
☒	☐	A statement on whether measurements were taken from distinct samples or whether the same sample was measured repeatedly
☐	☒	The statistical test(s) used AND whether they are one- or two-sided<br>*Only common tests should be described solely by name; describe more complex techniques in the Methods section.*
☒	☐	A description of all covariates tested
☒	☐	A description of any assumptions or corrections, such as tests of normality and adjustment for multiple comparisons
☒	☐	A full description of the statistical parameters including central tendency (e.g. means) or other basic estimates (e.g. regression coefficient) AND variation (e.g. standard deviation) or associated estimates of uncertainty (e.g. confidence intervals)
☒	☐	For null hypothesis testing, the test statistic (e.g. *F*, *t*, *r*) with confidence intervals, effect sizes, degrees of freedom and *P* value noted<br>*Give P values as exact values whenever suitable.*
☒	☐	For Bayesian analysis, information on the choice of priors and Markov chain Monte Carlo settings
☒	☐	For hierarchical and complex designs, identification of the appropriate level for tests and full reporting of outcomes
☒	☐	Estimates of effect sizes (e.g. Cohen's *d*, Pearson's *r*), indicating how they were calculated

Our web collection on statistics for biologists contains articles on many of the points above.

Software and code

Policy information about availability of computer code

Data collection	no software was used
Data analysis	Prism-GraphPad 9.0 was used for dose-response curve regression;<br>BWA, Bowtie2, LoFreq, LoFreq Filter tools from Galaxy.org were used for deep-seq analysis<br>Modelling studies were performed using the Maestro suite (Maestro version 13.4.134, MMshare Version 6.0.134, Release 2022-4

For manuscripts utilizing custom algorithms or software that are central to the research but not yet described in published literature, software must be made available to editors and reviewers. We strongly encourage code deposition in a community repository (e.g. GitHub). See the Nature Portfolio guidelines for submitting code & software for further information.

Data

Policy information about availability of data

All manuscripts must include a data availability statement. This statement should provide the following information, where applicable:

- Accession codes, unique identifiers, or web links for publicly available datasets
- A description of any restrictions on data availability
- For clinical datasets or third party data, please ensure that the statement adheres to our policy

The data that support the findings of this study are available from the corresponding author upon reasonable request.

Research involving human participants, their data, or biological material

Policy information about studies with human participants or human data. See also policy information about sex, gender (identity/presentation), and sexual orientation and race, ethnicity and racism.

Reporting on sex and gender	gender was determined by self-reporting
Reporting on race, ethnicity, or other socially relevant groupings	ethnicity is not reported
Population characteristics	age, diagnosis and treatment are reported in Table 1. These data were collected by the physician upon patients' informed consent
Recruitment	Patients were enrolled based on the following eligibility criteria: minimum 18 years of age; histological diagnosis of locally advanced or metastatic lung cancer; positivity for genetic rearrangements of ALK or ROS1, as identified on tissue samples by a clinically validated method (immunohistochemistry, FISH or NGS); radiological progression according to RECIST 1.1 criteria to treatment with specific ALK/ROS1 inhibitors in any treatment line. Patients pre-treated with chemotherapy, as well as patients with active brain metastases, were eligible
Ethics oversight	Comitato Etico Brianza

Note that full information on the approval of the study protocol must also be provided in the manuscript.

Field-specific reporting

Please select the one below that is the best fit for your research. If you are not sure, read the appropriate sections before making your selection.

☒ Life sciences ☐ Behavioural & social sciences ☐ Ecological, evolutionary & environmental sciences

For a reference copy of the document with all sections, see nature.com/documents/nr-reporting-summary-flat.pdf

Life sciences study design

All studies must disclose on these points even when the disclosure is negative.

Sample size	sample-size calculation was not performed
Data exclusions	no data were excluded from the analysis
Replication	all experiments were performed at least three times, using technical triplicates in each test.
Randomization	there is no randomization
Blinding	the people who performed data analysis were blinded to patients' clinical data

Reporting for specific materials, systems and methods

We require information from authors about some types of materials, experimental systems and methods used in many studies. Here, indicate whether each material, system or method listed is relevant to your study. If you are not sure if a list item applies to your research, read the appropriate section before selecting a response.

Materials & experimental systems

n/a	Involved in the study	
☐	☒	Antibodies
☐	☒	Eukaryotic cell lines
☒	☐	Palaeontology and archaeology
☒	☐	Animals and other organisms
☐	☒	Clinical data
☒	☐	Dual use research of concern
☒	☐	Plants

Methods

n/a	Involved in the study	
☒	☐	ChIP-seq
☒	☐	Flow cytometry
☒	☐	MRI-based neuroimaging

Antibodies

Antibodies used	phospho-ALK (Tyr1604) and total ALK (31F12) antibodies from Cell Signaling Technology.
Validation	they are commercial antibodies

Eukaryotic cell lines

Policy information about cell lines and Sex and Gender in Research

Cell line source(s)	Ba/F3 murine pro-B cell line were used
Authentication	they are a commercial cell line, authenticated by the provider
Mycoplasma contamination	the cells proved negative for mycoplasma
Commonly misidentified lines (See ICLAC register)	na

Clinical data

Policy information about clinical studies

All manuscripts should comply with the ICMJE guidelines for publication of clinical research and a completed CONSORT checklist must be included with all submissions.

Clinical trial registration	NCT06081270
Study protocol	clinicaltrials.gov
Data collection	Dec. 2021 - July 2023
Outcomes	PFS is calculated for each patient as the time from initiation of therapy with the current inhibitor at the time of liquid biopsy, to the date of progression or death from any cause.

Plants

Seed stocks	*Report on the source of all seed stocks or other plant material used. If applicable, state the seed stock centre and catalogue number. If plant specimens were collected from the field, describe the collection location, date and sampling procedures.*
Novel plant genotypes	*Describe the methods by which all novel plant genotypes were produced. This includes those generated by transgenic approaches, gene editing, chemical/radiation-based mutagenesis and hybridization. For transgenic lines, describe the transformation method, the number of independent lines analyzed and the generation upon which experiments were performed. For gene-edited lines, describe the editor used, the endogenous sequence targeted for editing, the targeting guide RNA sequence (if applicable) and how the editor was applied.*
Authentication	*Describe any authentication procedures for each seed stock used or novel genotype generated. Describe any experiments used to assess the effect of a mutation and, where applicable, how potential secondary effects (e.g. second site T-DNA insertions, mosiacism, off-target gene editing) were examined.*
